# Composite Films Based on Linear Polyethyleneimine Polymer and Starch or Polysaccharides from DDGS: Synthesis, Characterization, and Antimicrobial Studies

**DOI:** 10.3390/polym17040458

**Published:** 2025-02-09

**Authors:** Gonzalo Galaburri, Antonia Infantes-Molina, Cynthia M. Melian Queirolo, Andrea Mebert, María V. Tuttolomondo, Enrique Rodríguez-Castellón, Juan M. Lázaro-Martínez

**Affiliations:** 1 Facultad de Farmacia y Bioquímica, Departamento de Ciencias Químicas, Universidad de Buenos Aires, Ciudad Autónoma de Buenos Aires 1113, Argentina; ggonzagala@gmail.com (G.G.); cyn.m.melian@gmail.com (C.M.M.Q.); andreameb@gmail.com (A.M.); mvtuttolomondo@gmail.com (M.V.T.); 2 Instituto de Química y Metabolismo del Fármaco (IQUIMEFA-UBA-CONICET), CONICET—Universidad de Buenos Aires, Ciudad Autónoma de Buenos Aires 1113, Argentina; 3 Departamento de Química Inorgánica, Cristalografía y Mineralogía, Facultad de Ciencias, Instituto Interuniversitario en Biorrefinerías I3B, Universidad de Málaga, 29010 Málaga, Spain; ainfantes@uma.es

**Keywords:** DDGS, starch, polyethyleneimine, films, NMR, XPS, antimicrobial materials, mechanical properties

## Abstract

Different films were synthesized from starch or polysaccharides extracted from distillers dried grains with soluble (DDGS) in combination with different percentages of linear polyethyleneimine (PEI) hydrochloride polymer to assess the mechanical and antimicrobial properties of the resulting composites. Moreover, a simple method for the extraction of the polysaccharide content from DDGS is reported. The materials obtained were characterized by ATR-FTIR, NMR, and XPS spectroscopy, swelling capacity, and by organic elemental analysis. In particular, the stability of the film prepared with only DDGS in copper ion solutions was improved by the incorporation of PEI. ^13^C HRMAS NMR studies evidenced the incorporation of the PEI polymer in the new films. Moreover, the release of PEI molecules from the films was studied by ^1^H NMR experiments in D_2_O to explain the antimicrobial properties of the PEI-based films against *Staphylococcus aureus*, with the DDGS–10% PEI films being the most active surface. Furthermore, the incorporation of copper ions into the different films enhanced their antimicrobial activity. Additionally, the starch–10% PEI film exhibited good swelling capacity in deionized water (~1500%), which decreased with the addition of salts (~250%). Instead, the DDGS–10% PEI film showed low swelling capacity in deionized water (~80%), with this capacity increasing with the addition of salts (~250%). The mechanical properties of the films improved considerably when 3% PEI was used.

## 1. Introduction

Polysaccharides exhibit a remarkable diversity of shapes and configurations, endowing them with specific properties that can be strategically used in the design and synthesis of advanced materials. Their capacity to form three-dimensional networks and to establish specific interactions with other compounds facilitates the creation of materials with tailored mechanical, thermal, and chemical properties. Furthermore, polysaccharides possess the dual advantages of being biocompatible and biodegradable, making them ideal candidates for applications in medicine, the food industry, and sustainable packaging [1,2,3]. The versatility of polysaccharides has spurred extensive research to develop strategies of modifying and functionalizing them to customize their properties for specific needs, including thermal stability, mechanical resilience, or enabling the controlled release of active substances. In summary, the significance of polysaccharides in advanced materials’ chemistry lies in their role as versatile and sustainable building blocks, offering potential applications that range from the development of biomedical devices to ecological materials. This contribution plays a pivotal role in the generation of innovative solutions in the field of materials science and technology. Starch, a natural polymer derived from sources such as maize, potatoes, rice, wheat, and cassava, consists of glucose units linked by α-glycosidic linkages. Two starch structures are distinguished, i.e., amylose and amylopectin. Amylose features a linear structure with α-1→4 glycosidic linkages, while amylopectin has a branched structure with both α-1→4 and α-1→6 glycosidic linkages [4]. The ratio of amylose to amylopectin plays a crucial role in the gelatinization process of starch, influencing its crystallinity, granule size, and chemical properties [5,6]. Films with a higher amylose content tend to exhibit greater stiffness compared to that of those with a lower amylose content. From an application point of view, starch offers several advantages as a biopolymer. Its extraction is simpler and faster than that of other biopolymers, and its well-known chemical composition, coupled with its water solubility, facilitates its malleability [7,8].

In relation to biomasses, DDGS stand out as a prominent byproduct in the biodiesel production process. It provides high quantities of digestible fibers, including cellulose and hemicellulose (such as xylan), along with substantial protein content [9,10]. The diverse origins of DDGS, such as rice, wheat, barley, and corn, make it a versatile resource. In Argentina, the emphasis on corn, the most abundant source, underscores its significance in local biodiesel production. Impressively, for every ton of corn processed in a biodiesel plant, a remarkable 300 kg of DDGS are yielded, highlighting the generation efficiency of this byproduct. The surge in biodiesel production, driven by governmental initiatives to reduce reliance on gasoline, has concurrently amplified DDGS production in recent years. While DDGS finds various applications, its predominant use lies in animal fattening. This study aims to explore an alternative route for the isolation of polysaccharides from the digestible fibers within DDGS for the design of films [11,12,13].

On the other hand, the linear polyethyleneimine hydrochloride (PEI.HCl) polymer is a versatile semi-crystalline synthetic material with a positive net charge, which makes it soluble in water [14]. PEI can be easily synthesized, obtaining different molecular weights and structures, such as linear or branched [15]. In this work, a 22 kDa linear PEI was used [16]. This specific type of PEI has found applications in various fields, including gene delivery, adhesives, and antimicrobial agents [17,18,19,20,21]. Its positive net charge and water solubility also make it useful as a surfactant, stabilizing emulsions and enhancing particle dispersion in liquids. Moreover, linear PEI has been used for the synthesis of hydrogels with high capacity to load emerging pollutants like azo-dyes, antibiotics, and copper ions [22,23,24]. Furthermore, PEI has been studied for its antimicrobial properties, finding potential applications in antimicrobial coatings or materials. Additionally, PEI can be effectively blended with biopolymers such as cellulose, among others [18,19,25,26]. Blending PEI with biopolymers offers the opportunity to combine the unique properties of both materials, creating composite materials with tailored functionalities. The growing interest in developing antimicrobial materials through green synthesis represents a significant step towards addressing various challenges, including food preservation, reducing reliance on petroleum-based products, and mitigating microbial proliferation in undesired environments. One innovative approach to tackle these issues involves the creation of antimicrobial packaging materials, offering a sustainable solution with a wide range of applications [26,27]. This research endeavors to contribute to this field by evaluating the potential of polysaccharides extracted from DDGS [28] in comparison to starch [29]. These polysaccharides were then used to synthesize PEI-based composite materials to design antimicrobial films. Taking advantage of the properties of DDGS-derived polysaccharides and starch, this study aims to explore their efficacy in creating antimicrobial materials that can find applications in food packaging and other relevant contexts [30,31,32,33]. The proposed work aligns with the broader goal of promoting green synthesis methodologies, providing environmentally friendly alternatives while addressing the pressing challenges associated with microbial contamination. In this study, the potential of DDGS-derived polysaccharides as a key component in PEI-based composite films was assessed, opening new avenues for the development of sustainable and effective antimicrobial solutions in material science.

## 2. Materials and Methods

### 2.1. Materials

Starch, glycerol, sodium chloride, sodium sulfate, sodium hydroxide, copper(II) chloride, copper(II) sulfate, and deuterium oxide (D_2_O) (Sigma-Aldrich, St. Louis, MO, USA) were of analytic grade and used without further purification. DDGS from corn was supplied from ACABIO Bioethanol Plant (Córdoba, Argentina). The linear PEI.HCl polymer (22 kDa as free base and nitrogen content of 16.4%) was synthesized as described previously [16].

### 2.2. Characterization Techniques

Solid-state NMR and HRMAS NMR studies were done using a Bruker Avance-III HD spectrometer (Billerica, MA, USA) equipped with a 14.1 T narrow bore magnet operating at Larmor frequencies of 600.09 and 150.91 MHz for ^1^H and ^13^C, respectively. The films were studied by HRMAS NMR experiments by packing the sample swelled with D_2_O into a 4 mm ZrO_2_ HRMAS rotor with a 50 μL spherical insert (5 mg of film and 35 μL of D_2_O). The samples were spun at a magic angle spinning (MAS) rate of 4 kHz. The ^13^C NMR HRMAS spectra were recorded using single-pulse excitation experiments with high proton decoupling during acquisition (hpdec). An excitation pulse of 6 μs and a recycling time of 5 s were used. The SPINAL64 sequence (small phase incremental alternation with 64 steps) [34] was used for heteronuclear decoupling during acquisition. Chemical shifts (in ppm) are relative to Si(CH_3_)_4_. The number of scans was 2000 for all the samples (acquisition time ~2 h). For the ^13^C cross-polarization and magic angle spinning (^13^C CP-MAS) solid-state NMR experiments, the powdered samples were packed into 3.2 mm ZrO_2_ rotors and rotated at room temperature at MAS rates of 15 kHz using a 3.2 mm MAS probe. Glycine was used as an external reference compound for the recording of the ^13^C spectra and to set the Hartmann−Hahn matching condition in the CP-MAS experiments [35]. A contact time during CP of 2 ms and a recycling time of 5 s were used. The SPINAL64 sequence was used for heteronuclear decoupling during acquisition [34]. The number of scans was 4000 for all the samples (acquisition time ~4 h).

ATR-FTIR spectra were recorded on a Nicolet iS50 spectrometer (Thermo Scientific-Waltham, MA, USA) using a one-reflection diamond crystal. The XPS analysis was carried out with a Physical Electronics Versa-Pro II (Chanhassen, MI, USA) operating with a monochromatic X-ray source Al (Ka) of photons at 1486 eV under ultra-high vacuum using a pressure of 10^−6^ Pa. The organic elemental analysis was performed in a CE440 Elemental Analyser device (Coventry, UK). Tensile properties were studied in an Instron dynamometer model 5985 (Norwood, MA, USA) using a crosshead speed of 5 mm min^−1^ and a load cell of 0.1 kN. Maximum tensile strength, Young’s modulus, and elongation at break were obtained following ASTM D1708-18 standard recommendations [36]. Electron microscopy images were obtained on a Zeiss GeminiSEM 360 (Oberkochen, Germany) scanning electron microscope (SEM). The gravimetric method was employed to assess film swelling. The samples were immersed in solutions containing different salts for 24 h at room temperature. The salts used in the experiment were CuSO_4_, Na_2_SO_4_, CuCl_2_, and NaCl at concentrations of 50, 100, 250, and 500 mM. The following equation was employed to calculate the swelling percentage:

(1) $$Swelling\;\left(\%\right)=\frac{\left(Ws-Wd\right)\;}{Wd}\times 100$$

where *Wd* represents the initial weight of the dry film and *Ws* is the weight of the swollen film sample after the 24 h immersion period.

### 2.3. Synthesis of Starch–PEI Films

An aqueous solution (50 mL) containing 2 g of soluble starch, 1 g of glycerol, and 100 or 300 mg of PEI.HCl was heated to 65 °C for 60 min. The solution was then vacuum-filtered for 30 min to remove air bubbles trapped within. This step ensured a more uniform and bubble-free final product. Finally, the filtered solution was added to a silicone container and heated overnight at 60 °C. This step allows the solution to solidify and form the desired glass-like materials named starch–3% PEI or starch–10% PEI. Additionally, another film was prepared without PEI.HCl following the same procedure as that used for starch (Table 1). The average thickness of the starch films is shown in Table 1.

### 2.4. Synthesis of the DDGS–PEI Films

First, 8 g of crude DDGS was dispersed with 50 mL of deionized water for 40 min. The mixture was centrifuged and washed three times for 5 min at 4000× *g* and dried overnight at 65 °C. The material was then treated three times with ethyl acetate (50 mL), centrifuged at 4000× *g* for 5 min, and dried overnight at 65 °C, giving 6 g of washed DDGS (named as DDGS-W). Then, 1 g of DDGS-W was mixed with 12 mL of ethanol and 18 mL of a 0.8 M NaOH solution at 65 °C for 30 min. Subsequently, the dispersion was filtered, and the desired polysaccharide-rich DDGS (DDGS-P) was washed twice with water and used for the DDGS films without further treatment. Finally, 1 g of the wet DDGS-P was dispersed with 80 or 240 mg of PEI-HCl, 80 mg of glycerol, and 20 mL of deionized water at 65 °C for 1 h. After that, the mixture was vacuum-filtered for 30 min to remove air bubbles trapped in the solution. This step ensured a uniform and bubble-free final product. Lastly, the solution was placed in a silicone container overnight at 60 °C. This step allowed the solution to solidify and form the desired glass-like material named DDGS–PEI film. A film was also prepared without PEI.HCL following the same procedure (Table 2). The average thickness of the DDGS films is shown in Table 2.

### 2.5. Incorporation of Copper Ions into the Films

Strips of the different films were immersed in a 0.5 M solution of NaCl, Na_2_SO_4_, CuCl_2_, or CuSO_4_ for 5 min. The films were then removed from the solution and placed in an oven at 65 °C for 10 min. Finally, the films were cut into circular shapes (6.0 ± 0.5 mm).

### 2.6. Antimicrobial Activity Assays

To assess the antimicrobial activity, the inhibition halo in a solid culture medium was measured. *Staphylococcus aureus* (ATCC 3629) was used as the sensitive microorganism, and Trypto-Casein Soy Agar (TSA) was the solid medium. Briefly, a freshly prepared 1.5 × 10^8^ CFU mL^−1^ bacterial suspension (0.5 McFarland, as assessed by optical density at 600 nm) was homogeneously seeded on the agar surface. Circular film samples (6.0 ± 0.5 mm) were placed in the dish. The inhibition halo diameters were measured after overnight incubation at 37 °C. The samples were run in duplicate.

### 2.7. Releasing of PEI

PEI-based films (50 mg) were incubated with 600 μL of D_2_O for 72 h at room temperature. Afterward, the samples were centrifuged, and the supernatants were separated and placed in NMR tubes for analysis.

## 3. Results and Discussion

### 3.1. Spectroscopic Characterization of the Materials

**Figure 1:**
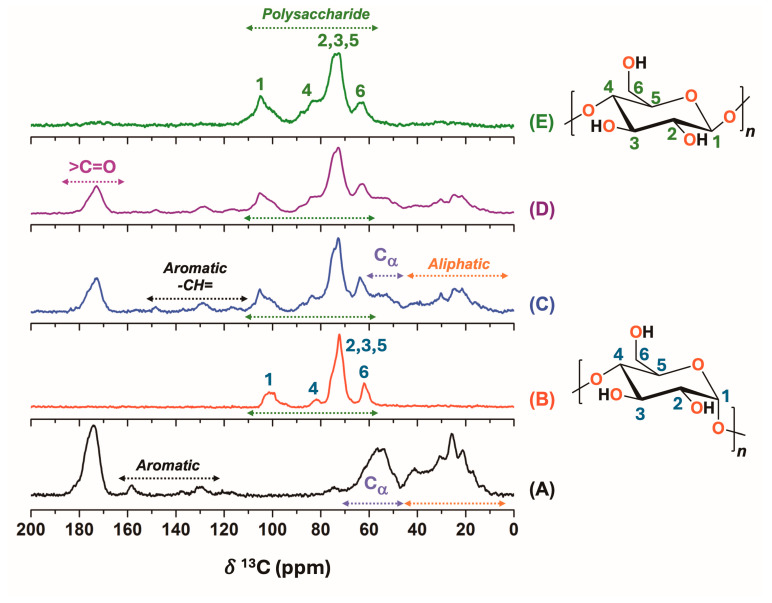
^13^C CP-MAS spectra (MAS rate: 15 kHz) for cow horn keratin (**A**), starch (**B**), native DDGS (**C**), DDGS-W (**D**), and DDGS-P samples (**E**).

The molecular composition of native DDGS and the isolation process of polysaccharide content from DDGS were studied by ^13^C CP-MAS solid-state NMR experiments (Figure 1). The ^13^C CP-MAS of the pristine DDGS powder sample showed carbon resonance signals associated with proteins, fatty acids, and polysaccharides (Figure 1C). The signal at a ^13^C chemical shift (*δ*^13^C) of 173 ppm corresponded to the carbonyl group of both the amide and ester groups present in proteins and triacylglycerols naturally present in the DDGS samples, respectively. Other resonance signals around 0–50 ppm 50–0 ppm were ascribed to the aliphatic region of some amino acids as well the methylene carbons of fatty acids of triacylglycerols. In particular, the polysaccharides content was clearly identified by the signals at *δ*^13^C~64, 74, 83, 104 ppm, with the anomeric carbon at 104 ppm, which is the characteristic signal of this biomolecule (Figure 1). Furthermore, some other minority signals were observed at *δ*^13^C of 116, 129, and 148 ppm, corresponding to the aromatic content of some amino acids like phenylalanine or tyrosine. As for the signals at 110–130 ppm, the contribution of unsaturated fatty acids or unsaturated triacylglycerols may be considered [37,38]. The ^13^C CP-MAS spectra of keratin isolated from cow’s horns [39,40] and commercial starch are shown in Figure 1 to illustrate the ^13^C resonance signal contributions from proteins and polysaccharides (Figure 1A,B).

The DDGS-W sample was also studied by solid-state NMR (Figure 1D). This sample was obtained by treating DDGS with ethyl acetate and water, showing no significant differences in the ^13^C CP-MAS spectra in comparison with those of the native DDGS sample. ^1^H NMR experiments in CDCl_3_ of the ethyl acetate fraction confirmed the presence of unsaturated triacylglycerols with some free fatty acids in the DDGS material [37,38]. The latter finding explained the carbon resonance contributions at *δ*^13^C of 110–130 and 173 ppm of unsaturated fatty acids naturally present in the DDGS material (Figure S1).

The alkaline treatment performed on the DDGS-W sample allowed isolating the DDGS-P material (Figure 1E), whose ^13^C CP-MAS spectrum only exhibited signals corresponding to polysaccharide content (*δ*^13^C = 60–110 ppm 110–60 ppm) [41,42,43,44], indicating that the other biomolecules, such as proteins and fatty acids, were removed from the DDGS structures by the alkaline treatment. It is noteworthy that even when the polysaccharide content was isolated from DDGS, some structural rearrangements in the biomolecule were detected, as evidenced by the changes in the signal intensities obtained in solid-state NMR spectra of the different DDGS samples (Figure 1C–E).

**Figure 2:**
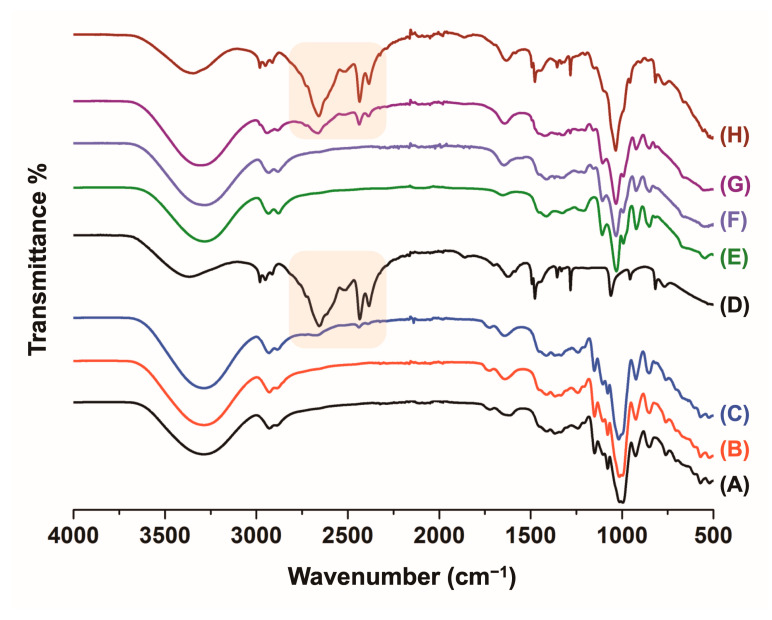
ATR-FTIR spectra for the films: starch (A), starch–3% PEI (B), starch–10% PEI (C), pristine linear PEI.HCl polymer (D), glycerol (E), DDGS (F), DDGS–3% PEI (G), and DDGS–10% PEI (H). The strong hydrogen bond interactions in the PEI.HCl polymer and the films are highlighted (2900–2300 cm*^−^*^1^).

The films were then characterized by ATR-FTIR spectroscopy (Figure 2). The starch ones showed the characteristic signals corresponding to the stretching vibrations of the -OH groups of the polysaccharide structure, which were observed in the frequency range of 3400–3000 cm⁻^1^ together with the stretching vibrations detected at 3000–2900 cm^−1^ (C-H), 1150–1100 cm^−1^ (C-O, C-C and C-O-H), and 1100–900 cm^−1^ (C-O-H bending) [45]. Some of the ATR-FTIR signals were overlapped with the contributing absorption bands of glycerol, which acts as a plasticizing agent for the films [46,47]. The ATR-FTIR spectrum of glycerol exhibited IR bands similar to those of starch, including the O-H stretching vibration at 3300 cm⁻^1^, the asymmetric and symmetric stretching vibration bands of -CH_2_- at 2934 and 2879 cm⁻^1^, respectively, and the bending vibration band of -CH_2_- at 1413 cm⁻^1^ (Figure 2E). Additionally, two C-O stretching vibration bands were observed at 1108 and 1030 cm⁻^1^, associated with the stretching modes of the secondary and primary alcohols, respectively. Moreover, the ATR-FTIR spectrum also revealed C-C stretchings, C-C-O symmetric stretchings, and O-H wagging absorption bands at 993, 851, and 674 cm⁻^1^, respectively (Figure 2) [48].

The linear PEI hydrochloride polymer exhibited intense absorption bands associated with its strong intermolecular hydrogen bonding network in the range of 2900–2300 cm⁻^1^ (Figure 2B–D) [22], which allowed its detection in the starch–10% PEI film. The strong hydrogen bonds were revealed by shifts in the N−H stretching frequency towards lower energy values. The strong IR signals from glycerol and starch masked other PEI-related signals in the films. The ATR-FTIR spectrum corresponding to the starch–3% PEI film did not display the IR absorption bands belonging to the PEI polymers mentioned above, due to their low concentration in this sample (Figure 2B).

The ATR-FTIR spectrum of the DDGS film without PEI displayed features similar to those of the starch materials (Figure 2F–H). The DDGS–3% PEI and DDGS–10% PEI films produced similar IR spectra, with marked differences in the intensity of the PEI signals at ~2900–2300 cm⁻^1^ for the DDGS–10% PEI film. Moreover, the stretching vibration of the C-N bonds associated with the PEI molecules was evident at 1250 cm^−1^ [22]. The band at 1680 cm⁻^1^ corresponded to water molecules adsorbed on the materials.

**Figure 3:**
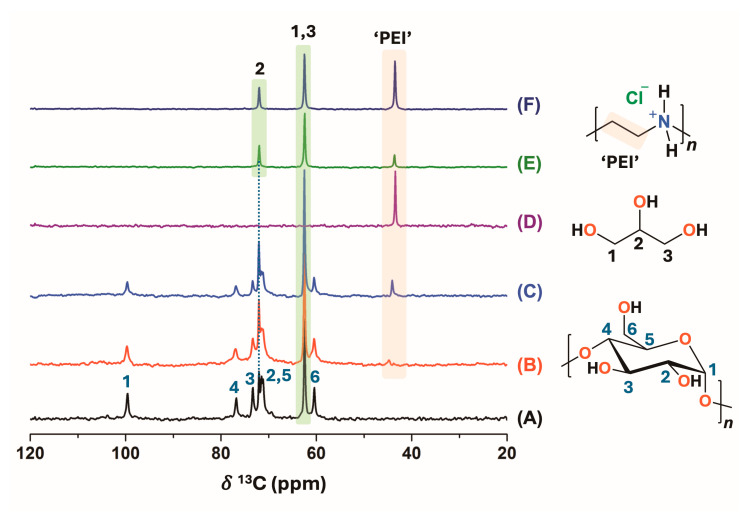
^13^C HRMAS NMR spectra (MAS rate: 4 kHz) for the different films obtained from starch (A), starch–3% PEI (B) or starch–10% PEI (C), DDGS–3% PEI (E) and DDGS–10% PEI swollen in D_2_O (F). The ^13^C NMR spectrum for PEI.HCl dissolved in D_2_O (D) is shown for comparison with those of the materials. The ^13^C HRMAS NMR experiments were acquired with direct-polarization techniques with ^1^H high-power decoupling during acquisition.

Furthermore, starch and DDGS films containing different PEI percentages were studied by ^13^C HRMAS NMR. In this case, the samples were swollen in D_2_O (Figure 3). The ^1^H HRMAS NMR spectra were also analyzed, but the *δ*^13^C of the elements that were present in both starch and DDGS films were better resolved in the ^13^C NMR scale. In this regard, the signals at *δ*^13^C of 60.4, 71.1, 73.6, 76.8, and 99.6 ppm were assigned to the hydrocarbon chain of starch as C_6_, C_2,5_, C_3_, C_4_, and C_1_ of the monomeric structure, respectively (Figure 3) [41,42]. Furthermore, the C_1,3_ and C_2_ of the glycerol molecules were observed at 62.5 and 72.0 ppm, respectively. These signals were superimposed with the carbon signals of the biopolymer in all the starch films due to the use of this compound as a plasticizer during the synthesis. Moreover, the incorporation of the PEI polymer in the synthesized films was confirmed by the presence of a peak at a *δ*^13^C = 43.5 ppm [16] both in the 3 and 10% PEI film formulations. The incorporation of PEI in starch–3% PEI could not be evidenced by ATR-FTIR. In fact, and considering the *δ*^13^C value, it could be assumed that PEI remained in the hydrochloride form in the films. The *δ*^13^C value for the PEI as a free-base form was found at ~51 ppm [22], allowing ^13^C HRMAS NMR studies the discrimination of the protonation state of the nitrogen atoms in the polymeric backbone inside the films (Figure 3).

As for DDGS materials, the polysaccharide content isolated from the DDGS-W sample was replaced by starch in the synthesis of the films. Unlike those of the starch films, the ^13^C HRMAS NMR spectra for the DDGS films showed only the signals corresponding to glycerol and PEI (Figure 3E,F). The polysaccharide signals could not be detected in this experiment, perhaps due to the low swelling degree of the polysaccharide in the DDGS–3% PEI and DDGS–10% PEI films. Again, in the DDGS–PEI films, PEI remained in the protonated form, as evidenced by the signal appearing at *δ*^13^C = 43.5 ppm. Considering that the ^13^C HRMAS NMR spectra were acquired with direct polarization, a semiquantitative determination of PEI content could be performed in the films and compared to that of glycerol. In this sense, films with 10% PEI retained more polymeric chains than those synthetized using 3% PEI did, as determined by the area of the PEI signal at 43.5 ppm. Furthermore, the comparison of the glycerol and PEI signal relative areas allowed determining that the content of PEI was higher in the DDGS–PEI film than in the starch–PEI films (Figure 3).

Likewise, the organic elemental analysis showed that PEI was successfully incorporated into the films, since a high content of nitrogen was found in the starch–PEI films (Table 3). Notably, the nitrogen fraction of all films increased in a directly proportional manner with the PEI concentration used in the synthesis, reflecting a consistent rise in nitrogen content. Moreover, low nitrogen content (0.2%) was detected in the DDGS after the alkaline treatment, according to the results obtained with the DDGS-P sample.

**Figure 4:**
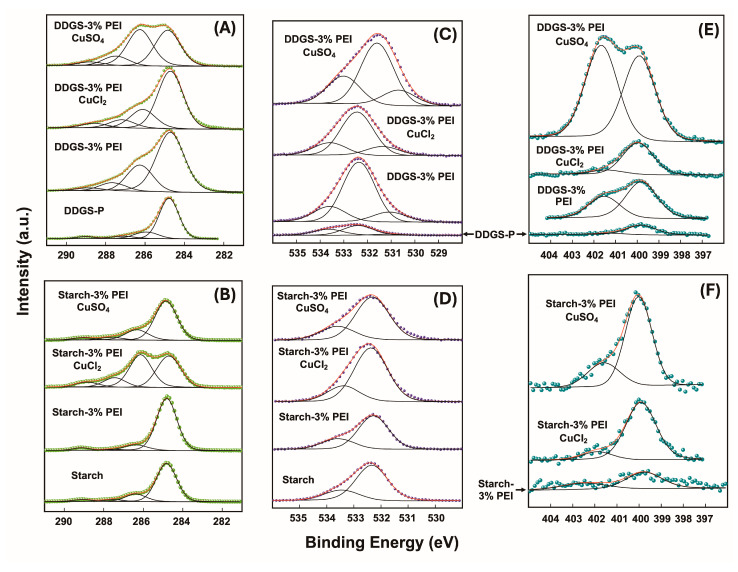
High resolution C 1s (**A**,**B**), O 1s (**C**,**D**), and N 1s (**E**,**F**) core level spectra for the indicated films and materials.

Finally, XPS studies were conducted to evaluate the surface chemical composition of the materials (Table 4) and to study the effects of the addition of copper ions in the PEI chain distribution within starch or DDGS films. The high resolution XPS spectra for the different materials and the results from the deconvolution studies are shown in Figure 4 and Tables S1–S10. The high resolution C 1s core level spectra for the different films containing starch or DDGS revealed four peaks originated by C-C/C-H bonds of adventitious carbon and PEI polymer chains (284.8 eV), C-N/C-O moieties from the PEI and polysaccharide structures (~286 eV), acetal (-HC(OR)O-R) and carbonyl carbons (~287 eV), and oxidized carbons belonging to carboxylic acid groups (~289 eV) [49]. The high resolution O 1s core level spectra for the starch films revealed two signals at around 532 and 533 eV corresponding to the oxygen atoms of the acetal and hydroxyl groups, respectively, with the hydroxyl groups belonging to both glycerol (plasticizer) and polysaccharide molecules (starch or DDGS-P material). For the DDGS films, the O 1s core level spectra showed three peaks assigned to the acetal, hydroxyl, and carbonyl groups at binding energies of around 533, 532, and 531 eV, respectively. Particularly, the presence of carbonyl groups in the C 1s and O 1s spectra in the DDGS films might be assigned to carboxylate moieties extracted together with the polysaccharide material from DDGS, in which a part of the triacylglycerol molecules remaining in the DDGS-P sample were hydrolyzed in the alkaline treatment. In the starch films, the C 1s signal detected at 289.1 eV was associated with some residual surface lipids [50], but no signals were observed at 531 eV in the O 1s spectrum due to their low content in the whole sample, as compared to that in the DDGS-W sample. These results reinforce the idea that in the DDGS-P sample used for the synthesis of the DDGS films, some carboxylic acids were present. Regarding the high resolution N 1s core level spectra, all the films displayed two contributions at 399.8 and 401.5 eV, which were associated with the non-protonated and protonated nitrogen atoms of the PEI structure, respectively [16,23,24]. The degree of protonation of the PEI structure was around 30–40% for the starch–PEI or DDGS–PEI films, as determined by the deconvolution of the N 1s spectra (Figure 4 and Tables S1–S10). As for the coordination of copper ions, both protonated and coordinated nitrogen sites were difficult to distinguish from one another due to the coincidence of the binding energy values for both chemical processes [16,51]. In this context, both coordinated/protonated versus non-protonated nitrogen contents were quite similar in the DDGS synthetized either with 3 or 10% PEI, confirming a higher density of PEI-coordinated segments in the surface of the materials with DDGS in comparison with those of starch. The coordination with CuCl_2_ showed a higher proportion of the non-protonated PEI structure in the surface and a lower amount of copper ions, as compared to those of films coordinated with CuSO_4_, demonstrating that copper ions produced a structural reorganization of the PEI moieties within the films (Table 4). In all the copper-loaded films, the Cu 2p core level spectra conducted at short acquisition times showed the typical Cu 2p_3/2_-Cu 2p_1/2_ spin orbit doublets together with the shake-up satellite signal corresponding to Cu^2+^ at around 934 and 942 eV, respectively (Figure S2) [51,52,53,54]. When the acquisition time for the Cu 2p XPS spectrum was longer, the signal-to-noise ratio was also improved, but the photoreduction to Cu^1+^ became evident due to the disappearance of the shake-up satellite signal (Figure S2) [16,51].

The surface elemental analysis also indicated a slight increase in the atomic nitrogen content when the amount of PEI employed for the sysnthesis of the films increased (Table 4); however, this does not represent the bulk polymer content, as PEI is distributed across films. Organic elemental analysis is always used to quantify the content of PEI polymers in the films, and this methodology showed that the films synthesized with 10% of PEI were the materials with the highest nitrogen composition (Table 3). Remarkably, the increased nitrogen content detected by XPS studies after the addition of copper ions demonstrated a change in the arrangement of PEI chains within the films. This finding indicates that PEI chains were relocated to the first atomic layers of the films during the incubation with copper ion solutions. In this sense, starch–3% PEI showed 0.52 N%, but after the incubation with copper ions, this value increased to 1.56 or 1.86 N% with CuCl_2_ or CuSO_4_, respectively. On the other hand, N% increased significantly (from 3.46 to 11.15) in DDGS–10% PEI after the addition of CuSO_4_. On the other hand, the surface copper content was similar in films with 3 or 10% PEI, being slightly higher in the DDGS–3% PEI and CuSO_4_ film (Table 4).

### 3.2. Mechanical, SEM, and Swelling Characterization

**Figure 5:**
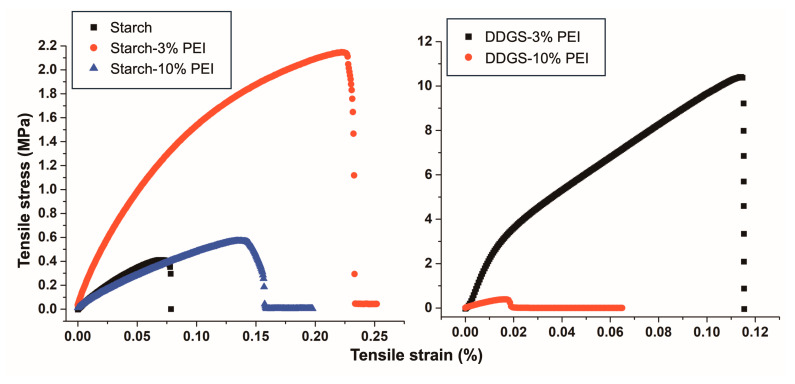
Mechanical behavior for starch, starch–PEI, and DDGS–PEI films with different PEI percentages.

The mechanical properties were evaluated on the different films to show the difference between starch and DDGS-P for the synthesis of films and the impact of the incorporation of PEI in the different materials (Table 5 and Figure 5). In general, starch matrices were less rigid than the DDGS matrices, as reflected by their lower Young’s modulus compared to that of the DDGS films (Table 5). The addition of 3% PEI enhanced the mechanical properties of both starch and DDGS films, while higher concentrations led to a decrease in Young’s modulus (Table 5). The starch–3% PEI film showed a higher elongation percentage at break point when compared to other films, demonstrating that the low content of PEI (3%) brought higher flexibility to the films. Additionally, the energy required to reach the breaking point was higher in the films containing 3% PEI, confirming the improvement in the mechanical strength of the films. The addition of PEI enhanced the tensile strength of starch and DDGS films with 3% PEI, which may be due to the strong hydrogen bonding interactions between PEI and the polysaccharide materials [55], which were higher in the DDGS film. Increasing PEI in the synthetic procedures to 10% led to a reduction of the tensile properties of starch and DDGS films (Figure 5). It is noteworthy that the use of commercial branched PEI to improve the mechanical properties of films in comparison with the linear forms of PEI has been reported [18,25,55,56,57]; however, in those works, neither information regarding the molecular weight nor the protonation state of the polymers were provided [58,59,60]. The mechanical properties of the pure DGGS films could not be evaluated because they were very easily broken before the tensile test could be performed.

**Figure 6:**
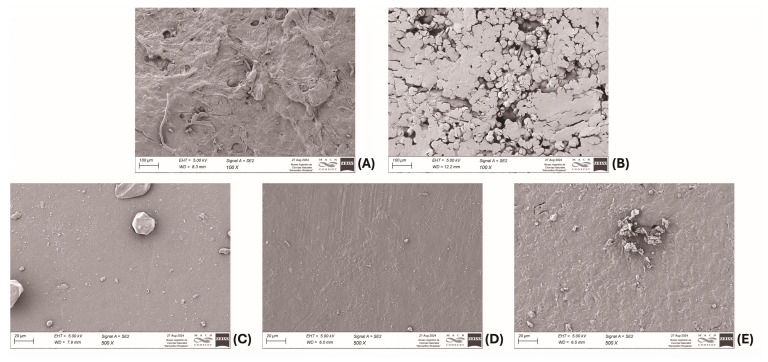
SEM images for DDGS–3% PEI (**A**) or DDGS–10% PEI (**B**), starch (**C**), and starch–3% PEI (**D**) or starch–10% PEI (**E**) films.

SEM images revealed changes in the surface structure of the films (Figure 6). DDGS–PEI films exhibited significant alterations in the surfaces of polymers bearing PEI, with DDGS–3% PEI having a more homogeneous and rougher surface as compared to that of DDGS–10% PEI, in which globular structures appeared on the surface. In contrast, starch and starch–PEI films displayed a similar homogenous and regular surface pattern. Particularly, the addition of PEI to the starch film produced a slight roughness on the surface, but not to the same degree as in the DDGS–PEI films, in which the changes were evident (Figure 6B). In general, the incorporation of PEI gave the films a rough appearance, which may explain the improvement in the mechanical properties obtained at low concentrations. Nevertheless, when PEI content was increased, the surfaces reduced their elongation capacity. Particularly, the irregular surface of the DDGS–10% PEI film explained the significant reduction of Young’s modulus and the maximum tensile strength.

**Figure 7:**
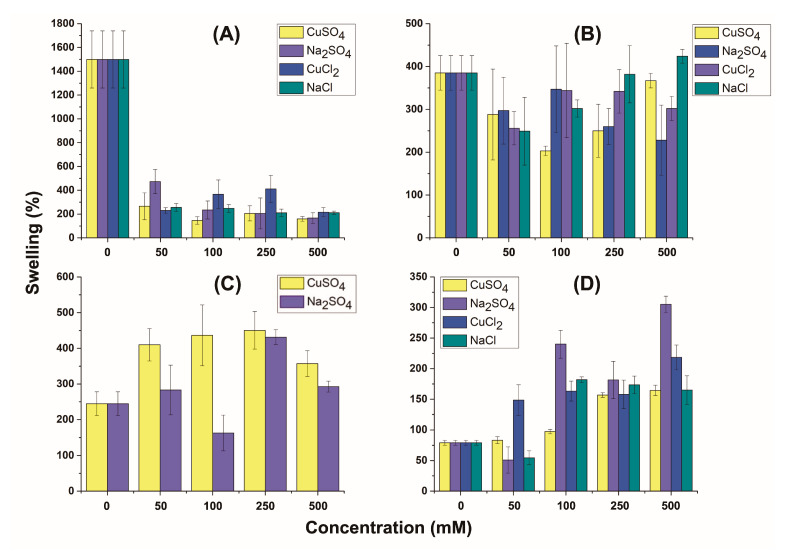
Swelling studies for the starch–3% PEI (**A**), starch–10% PEI (**B**), DDGS–3% PEI (**C**), and DDGS–10% PEI films (**D**). Error bars in the figures indicate the experimental standard error ± standard deviation.

Finally, the swelling degree of the different composite films was assessed in deionized water and different salt solutions (Figure 7). The swelling study allowed to determine the characteristics of the matrices for the preparation of new films. It also allowed to study whether the counterions that accompanied copper favored the stability of the material. The results determined which of the films were more hydrophilic, since a higher degree of swelling indicates greater interaction of the solution with the matrix of a film. Conversely, low swelling is observed when a film matrix contracts. These studies are important for studying the stability of the material against different solutions. This behavior is crucial for the stability of food packaging [29,31,61], avoiding contamination, or for the adsorption and detection of heavy metal ions or pollutants in contaminated waters [33,60]. Starch–3% PEI exhibited significant swelling in deionized water, which decreased markedly with the addition of salts (Figure 7A). Increasing concentrations of Na_2_SO_4_ and CuSO_4_ caused a decrease in the swelling capacity. Similarly, salts with chloride ions exhibited the same general trend: swelling decreased at low concentrations, then increased, and subsequently decreased again at high concentrations. For starch–10% PEI, no significant changes were observed with Na_2_SO_4_, NaCl, or CuCl_2_ solutions (Figure 7B). However, with CuSO_4_, swelling decreased to a minimum at 100 mM and then began to increase, reaching the highest value at 500 mM. These observations suggest that the interaction between starch–PEI films with different salts and concentrations had a significant impact on their swelling behavior, with specific patterns for each type of ion.

In the case of DDGS–3% PEI, the swelling capacity increased considerably in the CuSO_4_ solution and then decreased (Figure 7C). In contrast, the swelling decreased with 100 mM Na_2_SO_4_ and then rose abruptly to match the swelling observed with CuSO_4_. This initial decrease may be due to the cross-linking of sulfate ions with PEI chains, which reduced the swelling. Meanwhile, in the film with CuSO_4_, Cu^2+^ ions compete with SO_4_^2−^ ions for the nitrogen atoms of the PEI structure, and cross-linking does not occur. The DDGS–3% PEI film dissolved in the chloride solution; for this reason it could not be tested.

For DDGS–10% PEI, the lowest swelling was observed in deionized water, as compared to that of DDGS–3% PEI and the other materials (Figure 7D). With CuSO_4_, the swelling remained stable up to 100 mM and then increased to a maximum at 250 mM. In Na_2_SO_4_ solutions, a more abrupt drop in swelling was observed at 50 mM, followed by increases at 100 mM and 250 mM and another rise at 500 mM. It appears that SO_4_^2−^ ions crosslinked the PEI polymer chains at low concentrations [16], but a significant increase in the swelling was observed between 100 and 500 mM. This behavior was completely different from that observed in starch–10% PEI, indicating that other polar molecules isolated along with the polysaccharides (DDGS-P) increased the swelling capacity of the DDGS films. As for chloride salts, no significant changes were observed among the different concentrations tested, although a slight increase in swelling was noted with increasing salt concentrations.

### 3.3. Antimicrobial Properties

**Figure 8:**
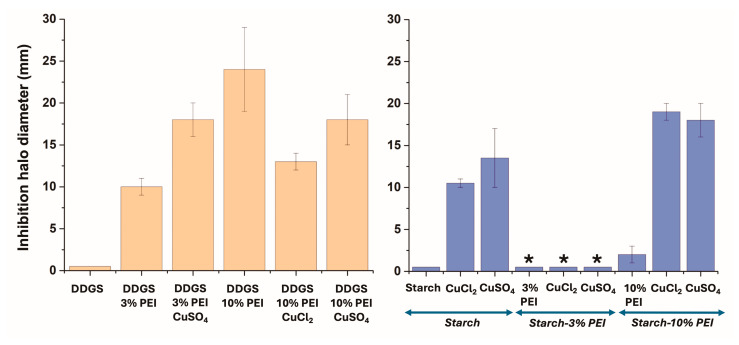
Inhibition halo in a solid medium against *S. aureus* for the indicated films. The ilms were based on DDGS (**left**) or starch (**right**). The materials indicated with an asterisk induced contact inhibition. Error bars in the figures indicate the experimental standard error ± standard deviation.

To explore the antimicrobial properties against *S. aureus*, the films were tested in a solid culture medium (Figure 8). The synthesized composite films were also loaded with copper ions, which can coordinate PEI polymers [16,62] and are known to have antimicrobial properties [63]. Thus, the aim of these experiments was to combine the effect of PEI polymers in disrupting the membrane of Gram-positive bacteria [17] and the incorporation of copper ions into the cells with the concomitant induction of oxidative stress [64]. *S. aureus* was selected from among the Gram-positive bacteria due to its relevance in infectious diseases and food poisoning related to the production and release of exotoxins in both humans and animals [65].

As expected, the starch film (without PEI or DDGS) did not show antimicrobial properties. The addition of 3% PEI (starch–3% PEI) yielded a surface without antimicrobial activity but causing contact inhibition (Figure 8). Moreover, for starch–10% PEI, a slight inhibition halo was observed (2 *±* 1 mm). Remarkably, the antimicrobial activity was enhanced with the inclusion of copper ions in the starch–PEI films, demonstrating synergy between PEI and copper ions. In other studies, starch has been used as a composite material with zinc oxide nanoparticles with antimicrobial applications, showing the potential of starch-based composites as antimicrobial materials [66,67]. However, the main advantage of starch–3% PEI lies in its capacity to induce contact inhibition. This property is particularly beneficial for protecting food and ensuring its safety [68,69,70]. Furthermore, with the addition of both copper salts, a considerable increase in the antimicrobial activity of the starch films was observed, with inhibition halo diameters of 10.5 *±* 0.5 and 13.5 *±* 3.5 mm for CuCl_2_ and CuSO_4_, respectively (Figure 8). It is considered that copper ions are gradually released from the surface of the different copper films. The main bactericidal mechanism reported for copper ions involves the generation of reactive oxygen species (ROS), which irreversibly damages membranes, while copper ions also degrade RNA and disrupt the membranes of enveloped viruses; against fungi, copper causes physical membrane deterioration and ion influx [63,64,71].

DDGS–3% PEI and DDGS–10% PEI films showed a significant increment in antimicrobial activity when compared to starch–PEI films (Figure 8). In this regard, the disposition of PEI chains in the DDGS film structures might allow for a higher release than in the starch films, explaining the greater inhibition halo diameters. Copper-loaded DDGS films without PEI could not be evaluated as they dissolved under the experimental conditions. The same phenomenon was observed with DDGS–3% PEI treated with CuCl_2_. However, DDGS–3% PEI treated with a CuSO_4_ solution remained stable as a film, likely due to the cross-linking effect of sulfate ions and PEI polymer chains [16]. In particular, upon incorporating CuSO_4_ into the DDGS–3% PEI film, an increase in the antimicrobial activity was observed due to a synergistic effect. Increasing the PEI concentration in the DDGS film resulted in an enhancement of antimicrobial activity. On the other hand, when copper ions were added into the DDGS–10% PEI film, a decrease in antimicrobial activity was observed for both salts. This behavior may be explained by the generation of more stable copper(II) complexes when PEI content was higher, avoiding the release of both components from the film. However, the DDGS–10% PEI film treated with CuSO_4_ presented higher activity than the film treated with CuCl_2_ did, showing that the high PEI–Cu ratio on the film surface observed in XPS studies played a crucial role in the antimicrobial activity (Figure 4 and Figure 8, and Table 4).

### 3.4. Release of PEI

**Figure 9:**
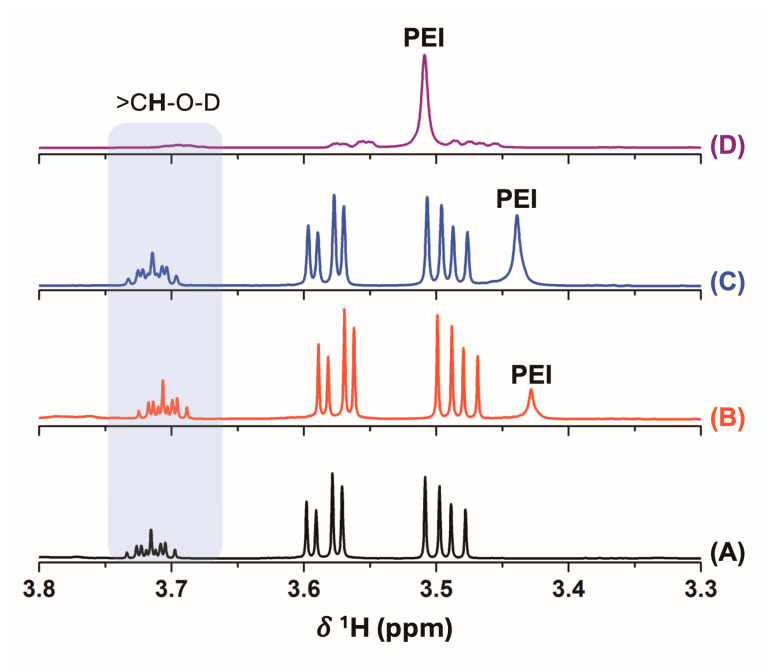
^1^H NMR spectra of the D_2_O solution obtained after the incubation of the starch–3% PEI (**A**), starch–10% PEI (**B**), DDGS–3% PEI (**C**), and DDGS–10% PEI (**D**) films after contact with 600 μL of D_2_O.

To correlate the antimicrobial activity with the release of PEI molecules from the films, different solution-state NMR experiments were conducted. The ^1^H NMR spectra for the D_2_O solutions incubated with the different films showed the typical signals from glycerol at *δ*^1^H values of 3.78, 3.58, and 3.55 ppm (Figure 9). The ^1^H NMR spectrum of glycerol is complex due to the rotational isomerism of the hydrocarbon chain in an aqueous solution independent of the temperature employed [72]. The typical signal at *δ*^1^H = 3.45 ppm for the methylene groups of the PEI structure was found in starch–10% PEI and DDGS–3% PEI (Figure 9). However, in the DDGS–10% PEI film, the PEI resonance signal was particularly shifted to 3.51 ppm. The increase in the pH values from 7.6 to 5.5 caused a superimposition of the latter signal with that of glycerol in the starch–PEI films. In this sense, at higher pH values, the PEI structure was deprotonated, thus changing its *δ*^1^H chemical. Furthermore, the starch–3% PEI film did not show the ^1^H signal of the PEI moieties (Figure 9A), showing that the polymer was not released from this surface and thus explaining the lack of antimicrobial activity (Table 3 and Figure 8). Probably, some interactions between PEI and starch molecules may be taking place in the films, which prevented their release from the surface and justified the absence of ^1^H resonance signals from both the polymer and biopolymer structures (Figure 9A).

Remarkably, the increment in the ^1^H NMR signal of PEI was in agreement with the increase of antimicrobial activity, as determined by the inhibition halo diameters in a solid culture medium (Figure 8). In all films, the resonance signal of PEI was compared to the signal of the hydrogen of the methine carbon of glycerol, allowing us to determine that the relative release of PEI as glycerol–PEI areas was 1:1.3, 1:9.7, 1:0, and 1:0.7, with inhibition halo diameters of 10 *±* 1, 24 *±* 5, 0, and 2 *±* 1 mm for the DDGS–3% PEI, DDGS–10% PEI, starch–3% PEI, and starch–10% PEI, respectively (Figure 8 and Figure 9). These results allowed relating the observed inhibition halos with the release of PEI polymeric chains from the different films, with the antimicrobial activity being increased for DDGS–10% PEI, from which the highest concentration of PEI was released.

## 4. Conclusions

In this work, the isolation of the polysaccharide content of DDGS was confirmed by a simple methodology using solid-state NMR experiments. Moreover, the polysaccharide material from DDGS or starch was effectively used in the synthesis of novel films. The incorporation of the linear PEI polymer into different surfaces was successfully done, as demonstrated by ATR-FTIR, XPS, and HRMAS NMR studies. The incorporation of PEI conferred the films antimicrobial properties and enhanced their mechanical properties in comparison with those of films synthesized using starch alone. Moreover, the SEM results were in agreement with the mechanical properties of the films, which were ascribed to the changes in the roughness of the films caused by the incorporation of PEI into the formulations. Swelling tests indicated reduced hydration capacity in the DDGS films compared to that in starch films. In addition, the mechanical properties of the DDGS films showed hardness higher than that of starch films.

The interaction between PEI and sulfate ions further contributed to a crosslinking effect on the films, which enhanced the stability of the DDGS–PEI films in aqueous solutions. Furthermore, the incorporation of copper to the starch film conferred it antimicrobial activity. However, the antimicrobial effect was stronger in the DDGS films with 3 or 10% of PEI in comparison to that of the film with starch or DDGS and glycerol alone. The use of both PEI and copper ions for the synthesis of the different films with DDGS or starch clearly enhanced their antimicrobial activity. Particularly, the starch–3% PEI and its copper films inhibited bacterial growth upon contact, which may be beneficial for the design of food packaging. The antimicrobial properties of the starch–PEI and DDGS–PEI materials were associated with the release of PEI polymer chains from the films, as confirmed by the ^1^H solution-state NMR studies in D_2_O.

There is a particular interest in finding alternatives to the petrochemical industry for food packaging. An ideal alternative has not yet been found, and this work explores the use of two abundant biopolymers that can be obtained from agroindustrial waste. Additionally, the addition of PEI and copper ions were studied to improve the performance of the films and provide new beneficial properties, such as antimicrobial properties for food preservation. In addition to being a renewable, abundant, and low-cost source, the use of DDGS from the agroindustry not only revalues wastes but also contributes to sustainability by reducing these polluting wastes that can decompose, while generating packaging considered more environmentally friendly than its fuel-based counterparts. In general, we have shown that the combination of DDGS or starch with PEI and copper ions is a promising strategy for this purpose. Furthermore, this work presents a new method for extracting polysaccharides from DDGS in a low-cost and environmentally friendly process, allowing us to continue studying new formulations and synthetic procedures for the generation of new materials.

## Figures and Tables

**Table 1:** Amounts of reagents used in the synthesis of starch films and the average film thickness.

Film	Starch (g)	Glycerol (g)	PEI.HCl (mg)	H_2_O (mL)	Film Thickness (mm ± SD)
Starch	2	1	-	50	0.615 ± 0.018
Starch–3% PEI	2	1	100	50	0.564 ± 0.004
Starch–10% PEI	2	1	300	50	0.401 ± 0.004

**Table 2:** Amounts of reagents used in the synthesis of DDGS films and the average film thickness.

Film	DDGS-P (g)	Glycerol (mg)	PEI.HCl (mg)	H_2_O (mL)	Film Thickness (mm ± SD)
DDGS	1	80	-	20	0.104 ± 0.012
DDGS–3% PEI	1	80	80	20	1.014 ± 0.030
DDGS–10% PEI	1	80	240	20	0.614 ± 0.133

**Table 3:** Organic elemental analysis of the different films and DDGS materials.

Material	% C	% H	% N
Starch	38.2	7.2	0
Starch–3% PEI	38.2	7.1	0.5
Starch–10% PEI	35.2	6.6	1.6
Native DDGS	46.8	7.1	4.3
DDGS-W	45.7	6.4	6.1
DDGS-P	33.6	8.0	0.2
DDGS–3% PEI	32.2	7.3	2.6
DDGS–10% PEI	30.2	7.4	7.7

**Table 4:** Organic surface atomic concentrations (C 1*s*, O 1*s*, Cl 2*p*, S 2*p*, N 1*s*, and Cu 2*p*) obtained by XPS.

Material	Atomic Concentration (%)					
	C 1*s*	O 1*s*	Cl 2*p*	S 2*p*	N 1*s*	Cu 2*p*
Starch	74.84	18.5	-	-	-	-
Starch–3% PEI	80.63	14.09	-	-	0.52	-
Starch–3% PEI + CuCl_2_	67.46	29.08	0.58	-	1.56	0.35
Starch–3% PEI + CuSO_4_	73.37	19.89	0.16	0.47	1.84	0.34
Starch–10% PEI	79.6	15.37	0.32	-	0.73	-
Starch–10% PEI + CuCl_2_	78.77	19.52	0.45	-	1.04	0.22
Starch–10% PEI + CuSO_4_	71.04	26.52	-	0.6	1.47	0.37
DDGS-P	86.6	12.4	-	-	1.0	-
DDGS–3% PEI	68.14	20.04	1.69	-	3.46	-
DDGS–3% PEI + CuCl_2_	71.4	18.24	0.9	-	2.4	0.38
DDGS–3% PEI + CuSO_4_	56.75	25.02	-	5.31	11.15	0.58
DDGS–10% PEI	64.31	19.12	2.8	-	7.9	-
DDGS–10% PEI + CuCl_2_	59.65	23.76	0.6	-	6.34	0.21
DDGS–10% PEI + CuSO_4_	54.75	25.28	-	5.64	11.82	0.41

**Table 5:** Mechanical properties of the different films.

Film	Young’s Modulus (MPa)	Maximum Tensile Strength (MPa)	Elongation at Break (%)	Energy to Break Point (J)
Starch	9.1 ± 0.2	0.4 ± 0.1	7.0 ± 0.9	0.00186 ± 0.00037
Starch–3% PEI	23 ± 4.2	2.1 ± 0.1	23.6 ± 4.2	0.02115 ± 0.00255
Starch–10% PEI	6.0 ± 0.7	0.4 ± 0.2	10.7 ± 3.9	0.00223 ± 0.00089
DDGS–3% PEI	231.6 ± 4.0	8.9 ± 2.1	11.1 ± 0.5	0.02762 ± 0.00036
DDGS–10% PEI	35.9 ± 4.4	0.3 ± 0.1	1.7 ± 0.3	0.005 ± 0.00130

## Data Availability

The original contributions presented in this study are included in the article/Supplementary Materials. Further inquiries can be directed to the corresponding authors.

## Supplementary Materials

The following supporting information can be downloaded at: https://www.mdpi.com/article/10.3390/polym17040458/s1, Figure S1: ^1^H and ^13^C NMR spectra (CDCl_3_) from the ethyl acetate fraction from DDGS; Figure S2: Cu 2*p* core level spectra at short (A) and long acquisition times (B); Figure S3: High-resolution C 1*s* (A), O 1*s* (B) and N 1*s* (C) core level spectra for the DDGS–3% PEI or DDGS–10% PEI films and CuSO_4_; Tables S1–S7: Fitting parameters from the XPS data of the starch films; Tables S8–S14: Fitting parameters from the XPS data of the DDGS films.

## References

[1] Paunonen S. (2013). Strength and Barrier Enhancements of Cellophane and Cellulose Derivative Films: A Review. Bioresources.

[2] Jiménez A., Fabra M.J., Talens P., Chiralt A. (2012). Edible and Biodegradable Starch Films: A Review. Food Bioproc Tech..

[3] Etale A., Onyianta A.J., Turner S.R., Eichhorn S.J. (2023). Cellulose: A Review of Water Interactions, Applications in Composites, and Water Treatment. Chem. Rev..

[4] Buléon A., Colonna P., Planchot V., Ball S. (1998). Starch Granules: Structure and Biosynthesis. Int. J. Biol. Macromol..

[5] Ashogbon A.O., Akintayo E.T. (2014). Recent Trend in the Physical and Chemical Modification of Starches from Different Botanical Sources: A Review. Starch—Stärke.

[6] Punia S., Kumar M., Siroha A.K., Kennedy J.F., Dhull S.B., Whiteside W.S. (2021). Pearl Millet Grain as an Emerging Source of Starch: A Review on Its Structure, Physicochemical Properties, Functionalization, and Industrial Applications. Carbohydr. Polym..

[7] Xiong H., Tang S., Tang H., Zou P. (2008). The Structure and Properties of a Starch-Based Biodegradable Film. Carbohydr. Polym..

[8] Peter Adigwe O., Egharevba H.O., Ochubiojo Emeje M. (2022). Starch: A Veritable Natural Polymer for Economic Revolution. Starch-Evolution and Recent Advances.

[9] Nuez Ortín W.G., Yu P. (2009). Nutrient Variation and Availability of Wheat DDGS, Corn DDGS and Blend DDGS from Bioethanol Plants. J. Sci. Food Agric..

[10] Han J., Liu K. (2010). Changes in Composition and Amino Acid Profile during Dry Grind Ethanol Processing from Corn and Estimation of Yeast Contribution toward DDGS Proteins. J. Agric. Food Chem..

[11] Chatzifragkou A., Kosik O., Prabhakumari P.C., Lovegrove A., Frazier R.A., Shewry P.R., Charalampopoulos D. (2015). Biorefinery Strategies for Upgrading Distillers’ Dried Grains with Solubles (DDGS). Process Biochem..

[12] Sirviö J.A., Visanko M., Ukkola J., Liimatainen H. (2018). Effect of Plasticizers on the Mechanical and Thermomechanical Properties of Cellulose-Based Biocomposite Films. Ind. Crops Prod..

[13] Gordobil O., Egüés I., Urruzola I., Labidi J. (2014). Xylan–Cellulose Films: Improvement of Hydrophobicity, Thermal and Mechanical Properties. Carbohydr. Polym..

[14] Lungu C.N., Diudea M.V., Putz M.V., Grudziński I.P. (2016). Linear and Branched PEIs (Polyethylenimines) and Their Property Space. Int. J. Mol. Sci..

[15] Jäger M., Schubert S., Ochrimenko S., Fischer D., Schubert U.S. (2012). Branched and Linear Poly(Ethylene Imine)-Based Conjugates: Synthetic Modification, Characterization, and Application. Chem. Soc. Rev..

[16] Lázaro-Martínez J.M., Rodríguez-Castellón E., Vega D., Monti G.A., Chattah A.K. (2015). Solid-State Studies of the Crystalline/Amorphous Character in Linear Poly (Ethylenimine Hydrochloride) (PEI·HCl) Polymers and Their Copper Complexes. Macromolecules.

[17] Gibney K.A., Sovadinova I., Lopez A.I., Urban M., Ridgway Z., Caputo G.A., Kuroda K. (2012). Poly(Ethylene Imine)s as Antimicrobial Agents with Selective Activity. Macromol. Biosci..

[18] Hernandez-Montelongo J., Lucchesi E.G., Nascimento V.F., França C.G., Gonzalez I., Macedo W.A.A., Machado D., Lancellotti M., Moraes A.M., Beppu M.M. (2017). Antibacterial and Non-Cytotoxic Ultra-Thin Polyethylenimine Film. Mater. Sci. Eng. C.

[19] Ayalew Z.M., Guo X., Zhang X. (2022). Synthesis and Application of Polyethyleneimine (PEI)-based Composite/Nanocomposite Material for Heavy Metals Removal from Wastewater: A Critical Review. J. Hazard. Mater. Adv..

[20] Taranejoo S., Liu J., Verma P., Hourigan K. (2015). A Review of the Developments of Characteristics of PEI Derivatives for Gene Delivery Applications. J. Appl. Polym. Sci..

[21] Forcato D.O., Fili A.E., Alustiza F.E., Lázaro Martínez J.M., Bongiovanni Abel S., Olmos Nicotra M.F., Alessio A.P., Rodríguez N., Barbero C., Bosch P. (2017). Transfection of Bovine Fetal Fibroblast with Polyethylenimine (PEI) Nanoparticles: Effect of Particle Size and Presence of Fetal Bovine Serum on Transgene Delivery and Cytotoxicity. Cytotechnology.

[22] Araque L.M., Pérez C.J., Infantes-Molina A., Rodríguez-Castellón E., Copello G.J., Lázaro-Martínez J.M. (2023). Linear PEI-Based Responsive Hydrogels: Synthesis and Characterization. J. Appl. Polym. Sci..

[23] Araque L.M., Infantes-Molina A., Rodríguez-Castellón E., Garro-Linck Y., Franzoni B., Pérez C.J., Copello G.J., Lázaro-Martínez J.M. (2024). Ionic Crosslinking of Linear Polyethyleneimine Hydrogels with Tripolyphosphate. Gels.

[24] Araque L.M., Fernández de Luis R., Fidalgo-Marijuan A., Infantes-Molina A., Rodríguez-Castellón E., Pérez C.J., Copello G.J., Lázaro-Martínez J.M. (2023). Linear Polyethyleneimine-Based and Metal Organic Frameworks (DUT-67) Composite Hydrogels as Efficient Sorbents for the Removal of Methyl Orange, Copper Ions, and Penicillin V. Gels.

[25] Zhang J., Han Y., Ben Z., Han T., Yin P. (2023). Effect of Branched Polyethyleneimine and Citric Acid on the Structural, Physical and Antibacterial Properties of Corn Starch/Chitosan Films. Int. J. Biol. Macromol..

[26] Fu L., Peng Y. (2017). Isocyanate-Functionalized Starch as Biorenewable Backbone for the Preparation and Application of Poly(Ethylene Imine) Grafted Starch. Monatshefte Für Chem.—Chem. Mon..

[27] Ates B., Koytepe S., Ulu A., Gurses C., Thakur V.K. (2020). Chemistry, Structures, and Advanced Applications of Nanocomposites from Biorenewable Resources. Chem. Rev..

[28] Xu W., Reddy N., Yang Y. (2009). Extraction, Characterization and Potential Applications of Cellulose in Corn Kernels and Distillers’ Dried Grains with Solubles (DDGS). Carbohydr. Polym..

[29] Dubey A., Irudhayaraj S., Jaiswal A., Uddin I., Ahmad I. (2023). Bio-Nanocomposites: A Next Generation Food Packaging Materials.

[30] Kang D., Li Y., Dai X., Li Z., Cheng K., Song W., Yu D.-G. (2024). A Soothing Lavender-Scented Electrospun Fibrous Eye Mask. Molecules.

[31] Perera K.Y., Jaiswal A.K., Jaiswal S. (2023). Biopolymer-Based Sustainable Food Packaging Materials: Challenges, Solutions, and Applications. Foods.

[32] Halonen N., Pálvölgyi P.S., Bassani A., Fiorentini C., Nair R., Spigno G., Kordas K. (2020). Bio-Based Smart Materials for Food Packaging and Sensors—A Review. Front. Mater..

[33] Zhao C., Liu G., Tan Q., Gao M., Chen G., Huang X., Xu X., Li L., Wang J., Zhang Y. (2023). Polysaccharide-Based Biopolymer Hydrogels for Heavy Metal Detection and Adsorption. J. Adv. Res..

[34] Fung B.M., Khitrin A.K., Ermolaev K. (2000). An Improved Broadband Decoupling Sequence for Liquid Crystals and Solids. J. Magn. Reson..

[35] Hartmann S.R., Hahn E.L. (1962). Nuclear Double Resonance in the Rotating Frame. Phys. Rev..

[36] (2018). Standard Test Method for Tensile Properties of Plastics by Use of Microtensile Specimens.

[37] Bella G., Rotondo A. (2020). Theoretical Prediction of 13C NMR Spectrum of Mixed Triglycerides by Mean of GIAO Calculations to Improve Vegetable Oils Analysis. Chem. Phys. Lipids.

[38] Wang X., Yang D., Gan L., Zhang H., Shin J., Lee Y., Jang Y., Lee K. (2015). Effect of Positional Distribution of Linoleic Acid on Oxidative Stability of Triacylglycerol Molecules Determined by ^1^H NMR. J. Am. Oil Chem. Soc..

[39] Ramos M.L.P., Rivas-Rojas P., Ascolani H., Cavallo M., Bonino F., de Luis R.F., Guerbi M.X., Michelini F., Bernal C., Lázaro-Martínez J.M. (2024). Flexible Keratin Hydrogels Obtained by a Reductive Method. Mater. Chem. Front..

[40] Galaburri G., Peralta Ramos M.L., Lázaro-Martínez J.M., Fernández de Luis R., Arriortua M.I., Villanueva M.E., Copello G.J. (2019). PH and Ion-Selective Swelling Behaviour of Keratin and Keratose 3D Hydrogels. Eur. Polym. J..

[41] Wang Y., Ma Y., Gao X., Wang Z., Zhang S. (2022). Insights into the Gelatinization of Potato Starch by in Situ 1H NMR. RSC Adv..

[42] Šoltýs A., Hronský V., Šmídová N., Olčák D., Ivanič F., Chodák I. (2019). Solid-State 1H and 13C NMR of Corn Starch Plasticized with Glycerol and Urea. Eur. Polym. J..

[43] Zhao H., Kwak J.H., Conrad Zhang Z., Brown H.M., Arey B.W., Holladay J.E. (2007). Studying Cellulose Fiber Structure by SEM, XRD, NMR and Acid Hydrolysis. Carbohydr. Polym..

[44] Idström A., Schantz S., Sundberg J., Chmelka B.F., Gatenholm P., Nordstierna L. (2016). 13C NMR Assignments of Regenerated Cellulose from Solid-State 2D NMR Spectroscopy. Carbohydr. Polym..

[45] Warren F.J., Gidley M.J., Flanagan B.M. (2016). Infrared Spectroscopy as a Tool to Characterise Starch Ordered Structure—A Joint FTIR–ATR, NMR, XRD and DSC Study. Carbohydr. Polym..

[46] Rajapaksha S.W., Shimizu N. (2021). Development and Characterization of Functional Starch-Based Films Incorporating Free or Microencapsulated Spent Black Tea Extract. Molecules.

[47] Wu Y., Luo X., Li W., Song R., Li J., Li Y., Li B., Liu S. (2016). Green and Biodegradable Composite Films with Novel Antimicrobial Performance Based on Cellulose. Food Chem..

[48] Armylisas A.H.N., Hoong S.S., Tuan Ismail T.N.M. (2024). Characterization of Crude Glycerol and Glycerol Pitch from Palm-Based Residual Biomass. Biomass Convers. Biorefin.

[49] Chen X., Wang X., Fang D. (2020). A Review on C1s XPS-Spectra for Some Kinds of Carbon Materials. Fuller. Nanotub. Carbon. Nanostructures.

[50] Hao Y., Chen Y., Li Q., Gao Q. (2019). Synthesis, Characterization and Hydrophobicity of Esterified Waxy Potato Starch Nanocrystals. Ind. Crops Prod..

[51] Crespi A.F., Zomero P.N., Pérez A.L., Brondino C.D., Molina A.I., Linck Y.G., Monti G.A., Fernández M.A., Rodríguez-Castellón E., Lázaro-Martínez J.M. (2023). Montmorillonite Materials with Paramagnetic Metal Complexes: Structural Studies and Catalytic Degradation of Emerging Pollutants. J. Environ. Chem. Eng..

[52] Lázaro Martínez J.M., Rodríguez-Castellón E., Sánchez R.M.T., Denaday L.R., Buldain G.Y., Campo Dall’ Orto V. (2011). XPS Studies on the Cu(I,II)–Polyampholyte Heterogeneous Catalyst: An Insight into Its Structure and Mechanism. J. Mol. Catal. A Chem..

[53] Moretti E., Molina A.I., Sponchia G., Talon A., Frattini R., Rodriguez-Castellon E., Storaro L. (2017). Low-Temperature Carbon Monoxide Oxidation over Zirconia-Supported CuO–CeO2 Catalysts: Effect of Zirconia Support Properties. Appl. Surf. Sci..

[54] Lázaro-Martínez J.M., Lombardo Lupano L.V., Piehl L.L., Rodríguez-Castellón E., Campo Dall’ Orto V. (2016). New Insights about the Selectivity in the Activation of Hydrogen Peroxide by Cobalt or Copper Hydrogel Heterogeneous Catalysts in the Generation of Reactive Oxygen Species. J. Phys. Chem. C.

[55] Zou D., Li X., Wu M., Yang J., Qin W., Zhou Z., Yang J. (2023). Schiff Base Synergized with Protonation of PEI to Achieve Smart Antibacteria of Nanocellulose Packaging Films. Carbohydr. Polym..

[56] Ojstršek A., Chemelli A., Osmić A., Gorgieva S. (2023). Dopamine-Assisted Modification of Polypropylene Film to Attain Hydrophilic Mineral-Rich Surfaces. Polymers.

[57] Chen J., Huang W., Chen Y., Zhou Z., Liu H., Zhang W., Huang J. (2022). Facile Preparation of Chitosan-Based Composite Film with Good Mechanical Strength and Flame Retardancy. Polymers.

[58] Wu H., Li J., Wu Y., Gao H., Guan Y. (2021). High-Performanced Hemicellulose Based Organic-Inorganic Films with Polyethyleneimine. Polymers.

[59] Panin S.V., Luo J., Buslovich D.G., Alexenko V.O., Berto F., Kornienko L.A. (2022). Effect of Transfer Film on Tribological Properties of Anti-Friction PEI- and PI-Based Composites at Elevated Temperatures. Polymers.

[60] Wang L., Wu Y., Li G., Xu H., Gao J., Zhang Q. (2020). Superhydrophobic N-Octadecylsiloxane (PODS)-Functionalized PDA-PEI Film as Efficient Water-Resistant Sensor for Ppb-Level Hexanal Detection. Chem. Eng. J..

[61] Kirchkeszner C., Petrovics N., Tábi T., Magyar N., Kovács J., Szabó B.S., Nyiri Z., Eke Z. (2022). Swelling as a Promoter of Migration of Plastic Additives in the Interaction of Fatty Food Simulants with Polylactic Acid- and Polypropylene-Based Plastics. Food Control.

[62] Torres D.I., Villanueva M.E., Lázaro-Martínez J.M., Copello G.J., Campo Dall’ Orto V. (2018). Calcium Alginate Beads Reinforced with Synthetic Oligomers, Linear Polyethylenimine and Cu(II): Structural Stability and Potential Applications. Cellulose.

[63] Yu J., Huang X., Ren F., Cao H., Yuan M., Ye T., Xu F. (2024). Application of Antimicrobial Properties of Copper. Appl. Organomet. Chem..

[64] Weaver L., Noyce J.O., Michels H.T., Keevil C.W. (2010). Potential Action of Copper Surfaces on Meticillin-Resistant Staphylococcus Aureus. J. Appl. Microbiol..

[65] Zhu Z., Hu Z., Li S., Fang R., Ono H.K., Hu D.-L. (2023). Molecular Characteristics and Pathogenicity of Staphylococcus Aureus Exotoxins. Int. J. Mol. Sci..

[66] Nafchi A.M., Alias A.K., Mahmud S., Robal M. (2012). Antimicrobial, Rheological, and Physicochemical Properties of Sago Starch Films Filled with Nanorod-Rich Zinc Oxide. J. Food Eng..

[67] Charoensri K., Rodwihok C., Wongratanaphisan D., Ko J.A., Chung J.S., Park H.J. (2021). Investigation of Functionalized Surface Charges of Thermoplastic Starch/Zinc Oxide Nanocomposite Films Using Polyaniline: The Potential of Improved Antibacterial Properties. Polymers.

[68] Rossi C., Chaves-López C., Serio A., Casaccia M., Maggio F., Paparella A. (2022). Effectiveness and Mechanisms of Essential Oils for Biofilm Control on Food-Contact Surfaces: An Updated Review. Crit. Rev. Food Sci. Nutr..

[69] Zhang H., Guo X., Tian L., Wang N., Li Y., Kushmaro A., Marks R., Sun Q. (2022). Antibiofilm Activity of 3,3′-Diindolylmethane on Staphylococcus Aureus and Its Disinfection on Common Food-Contact Surfaces. Food Sci. Hum. Wellness.

[70] Park K.M., Yoon S.-G., Choi T.-H., Kim H.J., Park K.J., Koo M. (2020). The Bactericidal Effect of a Combination of Food-Grade Compounds and Their Application as Alternative Antibacterial Agents for Food Contact Surfaces. Foods.

[71] Salah I., Parkin I.P., Allan E. (2021). Copper as an Antimicrobial Agent: Recent Advances. RSC Adv..

[72] Nishida Y., Aono R., Dohi H., Ding W., Uzawa H. (2023). 1H-NMR Karplus Analysis of Molecular Conformations of Glycerol under Different Solvent Conditions: A Consistent Rotational Isomerism in the Backbone Governed by Glycerol/Water Interactions. Int. J. Mol. Sci..

