# Supplementary material for: Composite Films Based on Linear Polyethyleneimine Polymer and Starch or Polysaccharides from DDGS: Synthesis, Characterization, and Antimicrobial Studies
Source: Polymers (Basel). 2025 Feb 9;17(4):458. doi: 10.3390/polym17040458 (PMC11858853; doi:10.3390/polym17040458)
Supplement: Supplementary file 1 [file polymers-17-00458-s001.zip › polymers-3443867-supplementary.pdf]

“Supplementary Material”

Composite films based on linear polyethyleneimine polymer and starch or polysaccharides from DDGS: synthesis, characterization and antimicrobial studies

Gonzalo Galaburri [1,2], Antonia Infantes-Molina [3], Cynthia M. Melian Queirolo [1,2], Andrea Mebert [1,2], María V. Tuttolomondo [1,2], Enrique Rodríguez-Castellón [3,*] and Juan M. Lázaro-Martínez [1,2,*]

Table of Contents:

Content	*Page*
Figure S1	2
Figure S2	3
Figure S3	4
Tables S1-S7	5
Tables S8-S14	8

(A)

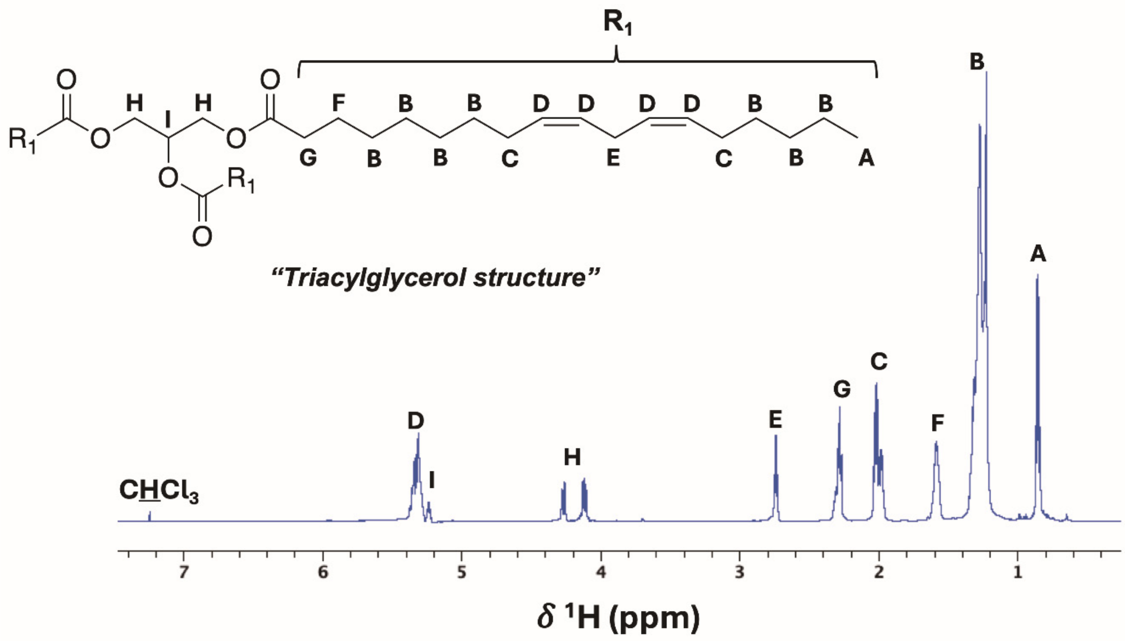

R1
H
I
H
F
B
B
D
D
D
D
B
B
G
B
B
C
E
C
B
A
"Triacylglycerol structure"
CHCl3
D
I
H
E
G
C
F
B
A
7
6
5
4
3
2
1
δ 1H (ppm)

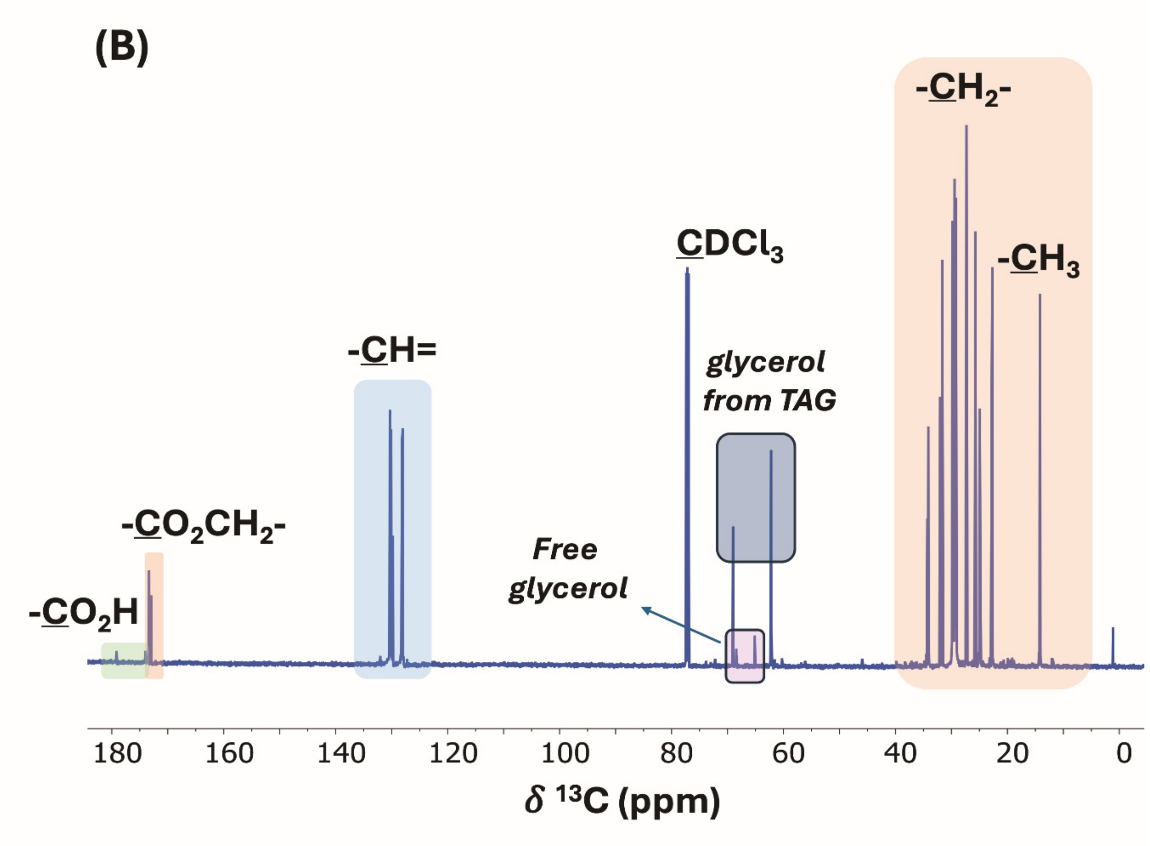

Figure S1. ^{1}H- and ^{13}C-NMR spectra ($CDCl_3$) from the ethyl acetate fraction from DDGS.

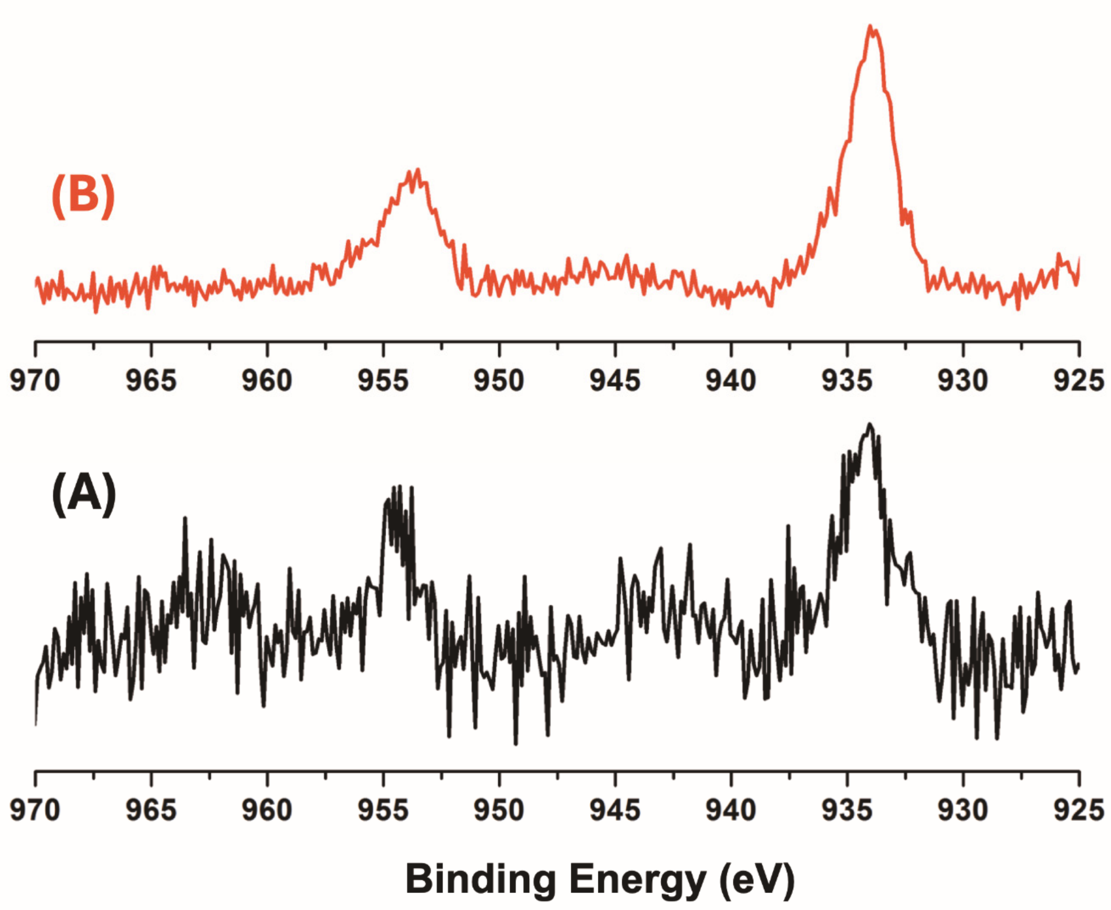

Figure S2. Cu 2*p* core level spectra at short (A) and long acquisition times (B).

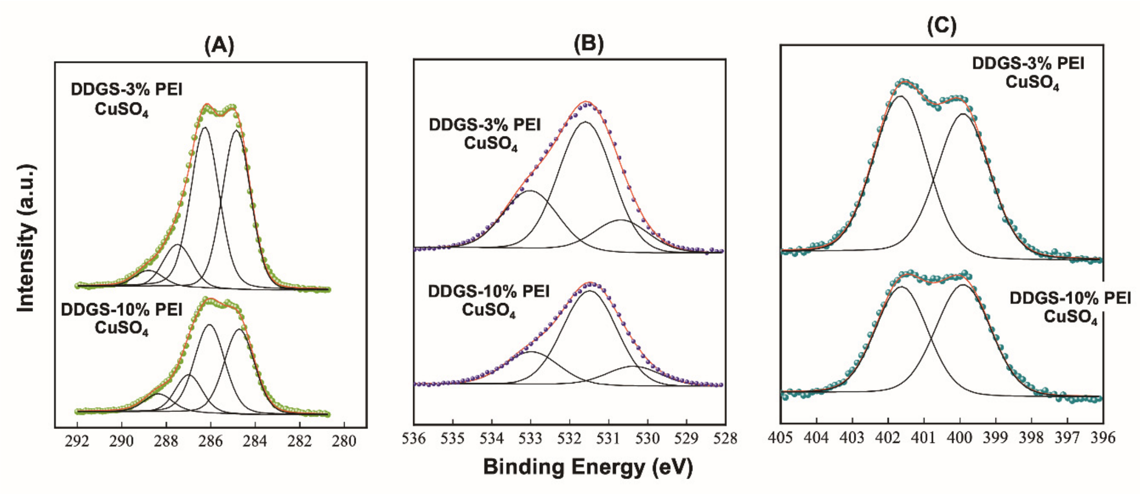

Figure S3. High resolution C 1*s* (A), O 1*s* (B) and N 1*s* (C) core level spectra for the DDGS-3% PEI or DDGS-10% PEI films and $CuSO_4$.

Table S1. Fitting parameters from C 1*s* and O 1*s* XPS data of the starch film.

C 1*s*								
**Band**	**Pos**	**PosSep**	**B_FWHM**	**FWHM**	**Height**	**%Gauss**	**Area**	**%Area**
1	284.83	0	1.2	1.2	10828	90	14498	76.36
2	286.38	1.56	1.23	1.23	2129	80	3065	16.14
3	287.51	2.69	1.22	1.22	532	90	724	3.81
4	289.08	4.25	1.22	1.22	540	100	699	3.68
**O 1*s***								
**Band**	**Pos**	**PosSep**	**B_FWHM**	**FWHM**	**Height**	**%Gauss**	**Area**	**%Area**
1	532.34	0	1.4	1.4	5639	82	8989	77.14
2	533.46	1.11	1.5	1.5	1668	100	2664	22.86

Table S2. Fitting parameters from C 1*s*, O 1*s* and N 1*s* XPS data of the starch-3% PEI film.

C 1*s*								
**Band**	**Pos**	**PosSep**	**B_FWHM**	**FWHM**	**Height**	**%Gauss**	**Area**	**%Area**
1	284.81	0	1.2	1.2	14730	89	19809	84.27
2	286.39	1.58	1.37	1.37	1572	80	2516	10.7
3	287.48	2.67	1.22	1.22	298	90	406	1.73
4	289.13	4.32	1	1	730	100	777	3.3
**O 1*s***								
**Band**	**Pos**	**PosSep**	**B_FWHM**	**FWHM**	**Height**	**%Gauss**	**Area**	**%Area**
1	532.28	0	1.31	1.31	5296	91	7684	75.64
2	533.59	1.31	1.4	1.4	1661	100	2475	24.36
**N 1*s***								
**Band**	**Pos**	**PosSep**	**B_FWHM**	**FWHM**	**Height**	**%Gauss**	**Area**	**%Area**
1	399.77	0	1.92	1.92	87	70	204	66.47
2	402.44	2.67	2.45	2.45	35	70	103	33.53

Table S3. Fitting parameters from C 1*s*, O 1*s* and N 1*s* XPS data of the starch-10% PEI film.

C 1*s*								
**Band**	**Pos**	**PosSep**	**B_FWHM**	**FWHM**	**Height**	**%Gauss**	**Area**	**%Area**
1	284.8	0	1.2	1.2	11297	89	15195	81.34
2	286.28	1.49	1.3	1.3	1743	80	2640	14.13
3	287.48	2.68	1.22	1.22	319	90	434	2.32
4	289	4.2	1.04	1.04	370	100	411	2.2
**O 1*s***								
**Band**	**Pos**	**PosSep**	**B_FWHM**	**FWHM**	**Height**	**%Gauss**	**Area**	**%Area**
1	532.36	0	1.44	1.44	4539	80	7625	84.47
2	533.59	1.23	1.4	1.4	941	100	1402	15.53
**N 1*s***								
**Band**	**Pos**	**PosSep**	**B_FWHM**	**FWHM**	**Height**	**%Gauss**	**Area**	**%Area**
1	399.72	0	1.75	1.75	85	80	173	59.71
2	401.73	2	1.77	1.77	57	80	117	40.29

Table S4. Fitting parameters from C 1*s*, O 1*s* and N 1*s* XPS data of the starch-3% PEI film and $CuCl_2$.

C 1*s*								
**Band**	**Pos**	**PosSep**	**B_FWHM**	**FWHM**	**Height**	**%Gauss**	**Area**	**%Area**
1	284.7	0	1.41	1.41	4445	99	6712	40.14
2	286.13	1.43	1.31	1.31	4602	80	7044	42.12
3	287.45	2.75	1.3	1.3	1336	90	1937	11.58
4	288.86	4.15	1.4	1.4	693	100	1030	6.16
**O 1*s***								
**Band**	**Pos**	**PosSep**	**B_FWHM**	**FWHM**	**Height**	**%Gauss**	**Area**	**%Area**
1	532.37	0	1.45	1.45	8556	90	13834	77.08
2	533.38	1.01	1.39	1.39	2541	80	4113	22.92
**N 1*s***								
**Band**	**Pos**	**PosSep**	**B_FWHM**	**FWHM**	**Height**	**%Gauss**	**Area**	**%Area**
1	399.92	0	1.62	1.62	298	80	562	87.35

2	401.88	1.96	1.61	1.61	43	80	81	12.65

Table S5. Fitting parameters from C 1*s*, O 1*s* and N 1*s* XPS data of the starch-3% PEI film and $CuSO_4$.

**C 1*s***								
**Band**	**Pos**	**PosSep**	**B_FWHM**	**FWHM**	**Height**	**%Gauss**	**Area**	**%Area**
1	284.85	0	1.31	1.31	11075	93	15924	70.98
2	286.33	1.48	1.35	1.35	2912	80	4575	20.39
3	287.63	2.78	1.3	1.3	765	90	1110	4.95
4	289.03	4.18	1.18	1.18	659	100	825	3.68
**O 1*s***								
**Band**	**Pos**	**PosSep**	**B_FWHM**	**FWHM**	**Height**	**%Gauss**	**Area**	**%Area**
1	532.32	0	1.45	1.45	6818	90	11024	74.05
2	533.6	1.29	1.51	1.51	2202	80	3864	25.95
**N 1*s***								
**Band**	**Pos**	**PosSep**	**B_FWHM**	**FWHM**	**Height**	**%Gauss**	**Area**	**%Area**
1	400.02	0	1.41	1.41	460	100	689	75.7
2	401.59	1.57	1.71	1.71	122	100	221	24.3

Table S6. Fitting parameters from C 1*s*, O 1*s* and N 1*s* XPS data of the starch-10% PEI film and $CuSO_4$.

**C 1*s***								
**Band**	**Pos**	**PosSep**	**B_FWHM**	**FWHM**	**Height**	**%Gauss**	**Area**	**%Area**
1	284.59	0	1.5	1.5	29640	83	51092	46.16
2	286.01	1.42	1.5	1.5	22267	80	38936	35.17
3	287.19	2.61	1.5	1.5	7852	100	12538	11.33
4	288.73	4.15	1.5	1.5	4769	86	8129	7.34
**O 1*s***								
**Band**	**Pos**	**PosSep**	**B_FWHM**	**FWHM**	**Height**	**%Gauss**	**Area**	**%Area**
1	531.32	0	1.62	1.62	8449	90	15287	14.98
2	532.51	1.18	1.62	1.62	39890	100	68902	67.53
3	533.68	2.36	1.62	1.62	9861	90	17842	17.49

N 1*s*								
**Band**	**Pos**	**PosSep**	**B_FWHM**	**FWHM**	**Height**	**%Gauss**	**Area**	**%Area**
1	399.83	0	1.8	1.8	1170	70	2562	66.84
2	401.76	1.93	1.95	1.95	535	70	1271	33.16

Table S7. Fitting parameters from C 1*s*, O 1*s* and N 1*s* XPS data of the starch-10% PEI film and $CuCl_2$.

C 1*s*								
**Band**	**Pos**	**PosSep**	**B_FWHM**	**FWHM**	**Height**	**%Gauss**	**Area**	**%Area**
1	284.84	0	1.5	1.5	41016	90	68606	61.87
2	286.37	1.53	1.5	1.5	16342	80	28576	25.77
3	287.61	2.76	1.5	1.5	4924	97	7956	7.18
4	289.01	4.17	1.5	1.5	3432	90	5741	5.18
**O 1*s***								
**Band**	**Pos**	**PosSep**	**B_FWHM**	**FWHM**	**Height**	**%Gauss**	**Area**	**%Area**
1	531.57	0	1.62	1.62	3844	90	6955	10.13
2	532.69	1.12	1.62	1.62	25921	100	44772	65.2
3	533.74	2.17	1.62	1.62	9808	100	16941	24.67
**N 1*s***								
**Band**	**Pos**	**PosSep**	**B_FWHM**	**FWHM**	**Height**	**%Gauss**	**Area**	**%Area**
1	400	0	1.77	1.77	786	70	1692	69.96
2	401.81	1.81	1.84	1.84	324	70	726	30.04

Table S8. Fitting parameters from C 1*s*, O 1*s* and N 1*s* XPS data of the DDGS-3% PEI film.

C 1*s*								
**Band**	**Pos**	**PosSep**	**B_FWHM**	**FWHM**	**Height**	**%Gauss**	**Area**	**%Area**
1	284.71	0	1.66	1.66	44809	80	86553	65.25
2	286.27	1.55	1.55	1.55	19607	100	32349	24.39
3	287.66	2.95	1.55	1.55	6095	100	10056	7.58
4	288.85	4.14	1.55	1.55	2181	94	3694	2.78
**O 1*s***								
**Band**	**Pos**	**PosSep**	**B_FWHM**	**FWHM**	**Height**	**%Gauss**	**Area**	**%Area**

1	531.07	0	1.66	1.66	6597	90	12240	12.62
2	532.36	1.29	1.6	1.6	39308	100	66947	69
3	533.6	2.53	1.6	1.6	9999	90	17840	18.39
**N 1*s***								
**Band**	**Pos**	**PosSep**	**B_FWHM**	**FWHM**	**Height**	**%Gauss**	**Area**	**%Area**
1	399.81	0	1.7	1.7	3624	80	7181	63.83
2	401.52	1.71	1.7	1.7	2147	90	4070	36.17

Table S9. Fitting parameters from C 1*s*, O 1*s* and N 1*s* XPS data of the DDGS-10% PEI film.

**C 1*s***								
**Band**	**Pos**	**PosSep**	**B_FWHM**	**FWHM**	**Height**	**%Gauss**	**Area**	**%Area**
1	284.69	0	1.66	1.66	30856	80	59602	57.42
2	286.21	1.52	1.55	1.55	18317	100	30222	29.11
3	287.78	3.09	1.55	1.55	6735	100	11112	10.7
4	288.89	4.2	1.55	1.55	1695	94	2871	2.77
**O 1*s***								
**Band**	**Pos**	**PosSep**	**B_FWHM**	**FWHM**	**Height**	**%Gauss**	**Area**	**%Area**
1	531.02	0	1.48	1.48	8446	90	13962	18.18
2	532.24	1.22	1.6	1.6	27890	100	47500	61.86
3	533.52	2.5	1.6	1.6	8591	90	15328	19.96
**N 1*s***								
**Band**	**Pos**	**PosSep**	**B_FWHM**	**FWHM**	**Height**	**%Gauss**	**Area**	**%Area**
1	399.78	0	1.7	1.7	7836	80	15528	73.08
2	401.43	1.66	1.7	1.7	3018	90	5721	26.92

Table S10. Fitting parameters from C 1*s*, O 1*s* and N 1*s* XPS data of the DDGS-3% PEI film and $CuSO_4$.

**C 1*s***								
**Band**	**Pos**	**PosSep**	**B_FWHM**	**FWHM**	**Height**	**%Gauss**	**Area**	**%Area**
1	284.85	0	1.5	1.5	26987	90	45140	41.39
2	285.96	1.42	1.5	1.5	27263	80	47672	43.71
3	287.19	2.64	1.5	1.5	7195	96	11724	10.75
4	288.49	3.94	1.5	1.5	2702	90	4519	4.14
**O 1*s***								
**Band**	**Pos**	**PosSep**	**B_FWHM**	**FWHM**	**Height**	**%Gauss**	**Area**	**%Area**

1	530.68	0	1.62	1.62	10173	90	18407	15.28
2	531.59	0.92	1.62	1.62	40620	99	70644	58.63
3	533.01	2.34	1.62	1.62	18207	100	31448	26.1
**N 1*s***								
**Band**	**Pos**	**PosSep**	**B_FWHM**	**FWHM**	**Height**	**%Gauss**	**Area**	**%Area**
1	399.9	0	1.75	1.75	8409	81	17098	48.44
2	401.65	1.75	1.77	1.77	9223	90	18199	51.56

Table S11. Fitting parameters from C 1*s*, O 1*s* and N 1*s* XPS data of the DDGS-3% PEI film and $CuCl_2$.

**C 1*s***								
**Band**	**Pos**	**PosSep**	**B_FWHM**	**FWHM**	**Height**	**%Gauss**	**Area**	**%Area**
1	284.71	0	1.5	1.5	43320	90	72458	63.4
2	286.12	1.41	1.5	1.5	14155	80	24752	21.66
3	287.18	2.47	1.5	1.5	6259	80	10945	9.58
4	288.59	3.88	1.5	1.5	3507	80	6133	5.37
**O 1*s***								
**Band**	**Pos**	**PosSep**	**B_FWHM**	**FWHM**	**Height**	**%Gauss**	**Area**	**%Area**
1	531.36	0	1.62	1.62	5947	90	10760	14.64
2	532.44	1.08	1.61	1.61	28497	100	48702	66.25
3	533.58	2.22	1.62	1.62	8133	100	14048	19.11
**N 1*s***								
**Band**	**Pos**	**PosSep**	**B_FWHM**	**FWHM**	**Height**	**%Gauss**	**Area**	**%Area**
1	399.96	0	1.65	1.65	3137	90	5772	87.53
2	401.4	1.44	1.79	1.79	413	90	822	12.47

Table S12. Fitting parameters from C 1*s*, O 1*s* and N 1*s* XPS data of the DDGS-10% PEI film and $CuSO_4$.

**C 1*s***								
**Band**	**Pos**	**PosSep**	**B_FWHM**	**FWHM**	**Height**	**%Gauss**	**Area**	**%Area**
1	284.72	0	1.6	1.6	14453	83	26574	37.51
2	286.06	1.34	1.6	1.6	15082	80	28130	39.71
3	286.99	2.27	1.4	1.4	6427	80	10489	14.81
4	288.35	3.63	1.5	1.5	3099	70	5654	7.98
**O 1*s***								

Band	Pos	PosSep	B_FWHM	FWHM	Height	%Gauss	Area	%Area
1	530.36	0	1.62	1.62	6185	90	11192	13.89
2	531.48	1.13	1.62	1.62	29708	100	51315	63.69
3	532.98	2.63	1.62	1.62	10181	94	18068	22.42
**N 1*s***								
**Band**	**Pos**	**PosSep**	**B_FWHM**	**FWHM**	**Height**	**%Gauss**	**Area**	**%Area**
1	399.9	0	1.81	1.81	6459	90	13011	52.4
2	401.4	1.72	1.7	1.7	6230	90	11821	47.6

Table S13. Fitting parameters from C 1*s*, O 1*s* and N 1*s* XPS data of the DDGS-10% PEI film and $CuCl_2$.

**C 1*s***								
**Band**	**Pos**	**PosSep**	**B_FWHM**	**FWHM**	**Height**	**%Gauss**	**Area**	**%Area**
1	284.70	0	1.5	1.5	43320	90	72458	63.2
2	286.22	1.41	1.5	1.5	14155	80	24752	21.8
3	287.19	2.47	1.5	1.5	6259	80	10945	9.6
4	288.57	3.88	1.5	1.5	3507	80	6133	5.4
**O 1*s***								
**Band**	**Pos**	**PosSep**	**B_FWHM**	**FWHM**	**Height**	**%Gauss**	**Area**	**%Area**
1	531.34	0	1.62	1.62	5947	90	10760	14.64
2	532.41	1.07	1.61	1.61	28497	100	48702	67.25
3	533.54	2.21	1.62	1.62	8133	100	14048	18.11
**N 1*s***								
**Band**	**Pos**	**PosSep**	**B_FWHM**	**FWHM**	**Height**	**%Gauss**	**Area**	**%Area**
1	399.93	0	1.65	1.65	3137	90	5772	87.5
2	401.1	1.43	1.79	1.79	413	90	822	12.5

Table S14. Fitting parameters from C 1*s*, O 1*s* and N 1*s* XPS data of the DDGS-P material.

**C 1*s***								
**Band**	**Pos**	**PosSep**	**B_FWHM**	**FWHM**	**Height**	**%Gauss**	**Area**	**%Area**
1	284.78	0	1.15	1.15	10156	93	12884	76.8
2	285.94	1.16	1.4	1.4	1648	100	2455	14.64
3	287.12	2.34	1.4	1.4	587	100	875	5.22
4	289.01	4.23	1	1	511	94	561	3.35

O 1*s*								
**Band**	**Pos**	**PosSep**	**B_FWHM**	**FWHM**	**Height**	**%Gauss**	**Area**	**%Area**
1	531.11	0	1.34	1.34	233	90	350	5.89
2	532.39	1.29	1.48	1.48	2118	95	3419	57.57
3	533.66	2.55	1.5	1.5	1297	90	2169	36.53
**N 1*s***								
**Band**	**Pos**	**PosSep**	**B_FWHM**	**FWHM**	**Height**	**%Gauss**	**Area**	**%Area**
1	399.81	0	1.63	1.63	157	80	299	88.71
2	401.3	1.48	1.63	1.63	21	90	38	11.29
